# Glycogen synthase kinase 3β inhibition synergizes with PARP inhibitors through the induction of homologous recombination deficiency in colorectal cancer

**DOI:** 10.1038/s41419-021-03475-4

**Published:** 2021-02-15

**Authors:** Ning Zhang, Yu-Nan Tian, Li-Na Zhou, Meng-Zhu Li, Hua-Dong Chen, Shan-Shan Song, Xia-Juan Huan, Xu-Bin Bao, Ao Zhang, Ze-Hong Miao, Jin-Xue He

**Affiliations:** 1 Division of Anti-Tumor Pharmacology, State Key Laboratory of Drug Research, Shanghai Institute of Materia Medica, Chinese Academy of Sciences, Shanghai, 201203 China; 2 University of Chinese Academy of Sciences, No.19A Yuquan Road, Beijing, 100049 China; 3 Department of Medicinal Chemistry, CAS Key Laboratory of Receptor Research, Shanghai Institute of Materia Medica, Chinese Academy of Sciences, Shanghai, 201203 China

**Keywords:** Cancer therapeutic resistance, Targeted therapies

## Abstract

Monotherapy with poly ADP-ribose polymerase (PARP) inhibitors results in a limited objective response rate (≤60% in most cases) in patients with homologous recombination repair (HRR)-deficient cancer, which suggests a high rate of resistance in this subset of patients to PARP inhibitors (PARPi). To overcome resistance to PARPi and to broaden their clinical use, we performed high-throughput screening of 99 anticancer drugs in combination with PARPi to identify potential therapeutic combinations. Here, we found that GSK3 inhibitors (GSK3i) exhibited a strong synergistic effect with PARPi in a panel of colorectal cancer (CRC) cell lines with diverse genetic backgrounds. The combination of GSK3β and PARP inhibition causes replication stress and DNA double-strand breaks, resulting in increased anaphase bridges and abnormal spindles. Mechanistically, inhibition or genetic depletion of GSK3β was found to impair the HRR of DNA and reduce the mRNA and protein level of BRCA1. Finally, we demonstrated that inhibition or depletion of GSK3β could enhance the in vivo sensitivity to simmiparib without toxicity. Our results provide a mechanistic understanding of the combination of PARP and GSK3 inhibition, and support the clinical development of this combination therapy for CRC patients.

## Introduction

Poly (ADP-ribose) polymerases (PARP) are important DNA repair enzymes^1^. PARP inhibitors (PARPi) including olaparib, niraparib, rucaparib, and talazoparib have been approved as monotherapy for patients with *BRCA1/2*-mutated ovarian cancers, breast cancer, pancreatic cancer, and prostate cancer^2,3^. Despite promising clinical results, as with other targeted drugs, the efficacy of PARPi is limited by the drug resistance. Only a fraction of *BRCA1/2* mutation carriers responded to PARPi, and even those who responded subsequently developed resistance and relapsed^4,5^. For example, only 6% of organoids generated from high-grade serous ovarian cancers (HGSC) were sensitive to olaparib, although a high number of patients were hypothesized to have homologous recombination (HR) defective tumors by genomic analysis^6^. Furthermore, the promise of PARPi in the management of BRCA1/2-deficient cancers is tempered by the fact that HR-proficient tumors do not respond to these agents. Thus, the development of strategies to selectively impair HR in cancer cells and subsequently sensitize the PARPi resistance of BRCA-deficient cancers and HR-proficient cancers to PARP inhibition may provide new clinical applications. In this regard, drug combination approaches have been designed and evaluated in preclinical and early clinical trials^7^. Currently, the combination of olaparib and bevacizumab has been approved for patients with advanced ovarian cancer^8^.

PARPi are thought to trap the PARP1/2 enzymes at the site of DNA damage, leading to replication-induced DNA damage that requires BRCA1/2-dependent homologous recombination repair (HRR)^9^. Therefore, DNA-damaging agents and molecular inhibitors that target DNA damage response pathways, such as ATR and Chk1, are expected to enhance the antitumor effect of PARPi^10,11^. PARPi in combination with other targeted therapies that are capable of disrupting HRR have also shown promising results in preclinical studies^7^. However, clinical studies showed that PARPi in combination with cytotoxic chemotherapies (such as topotecan, cisplatin, gemcitabine, and temozolomide) had limited clinical efficacy and high toxicity^12–15^. Therefore, new combination strategies are needed to improve the efficacy and alleviate the toxicity of combination therapy.

Clinical studies showed that more than 40% of BRCA1/2-deficient patients failed to respond to PARPi, which meant a high rate of de novo resistance to PARP inhibition even among in *BRCA*-mutated tumors^4,5^. Previous data revealed that PARPi including olaparib, niraparib, and simmiparib induced mild synthetic lethality in human breast cancer, HCC1937 (BRCA1-deficient), and colorectal cancers (CRC), HCT-15 (BRCA2-deficient), cells in vitro, and the antitumor activity was limited in mouse xenograft models^16–18^. Thus, HCC1937 and HCT-15 cells serve as model cell lines for de novo resistance to PARP inhibition. Specially, the majority of studies focused on a specific drug and PARP inhibition-induced alterations of its efficacy in BRCA-proficient cancer cells. As such, relatively little is known about the BRCA1/2 deficiency on PARPi-based combination and how PARPi alters the efficacy of a broad spectrum of drugs.

Glycogen synthase kinase (GSK3), a serine/threonine-protein kinase with two functionally distinct isoforms, α and β, was discovered in the context of glycogen metabolism and has emerged as a ubiquitous regulator of multiple signaling pathways^19,20^. Historically, GSK3β has been thought of as a potential tumor suppressor due to its regulatory effect in the Wnt/β-catenin pathway^21^. However, large and increasing bodies of published data over the past decade have demonstrated that GSK3β is a positive regulator of cancer cell proliferation and survival in multiple tumor types^22^. CRC cells also displayed aberrant GSK3β expression and activity^23–25^. Direct pharmacologic inhibition of GSK3β singling is, therefore, considered an attractive clinical strategy for these diseases^22^. A large number of GSK3 inhibitors (GSK3i) have entered clinical trials and several patent applications have been filed and/or granted^26^. Unfortunately, GSK3i have shown limited benefits, as monotherapy, in preclinical and clinical studies^27–31^. However, they appeared to be more effective when combined with other drugs^28,32–35^.

In this study, new combinatorial therapy strategies were investigated to improve the anticancer efficacy in *BRCA*-mutated cells with primary PARPi resistance. We first performed a preliminary screening of 99 anticancer drugs in combination with the PARPi, olaparib, and niraparib, in HCC1937 or HCT-15 cells. The results revealed that inhibition of PARP partly affected the cellular sensitivity to a panel of oncological drugs and kinase inhibitors. Among these agents, GSK3i exhibited the best synergistic effect with PARPi in BRCA2-deficient HCT-15 cells. Moreover, the data showed that the PARPi, simmiparib, acted synergistically with the GSK3i, CHIR99021 HCl, and LY2090314, in a panel of BRCA-proficient CRC cells. These results indicated that a combination of GSK3i and PARPi may serve as a new therapeutic strategy for CRC patients.

## Materials and methods

### Antibodies and chemicals

Simmiparib was provided by Dr. Ao Zhang and prepared as described previously^36^. Olaparib, talazoparib, niraparib, rucaparib, irinotecan, adriamycin, etoposide, hydroxyurea, and the 99 inhibitors (listed in Table S1) used for combination screening were purchased from Selleck Chemicals (Houston, TX, USA). Antibodies against Mre11 (sc-5858), CtIP (sc-271339), BRCA1 (sc-642), Rad52 (sc-8530), and Rad51 (sc-8349) were from Santa Cruz Biotechnology (Santa Cruz, CA, USA). Antibodies against γ-H2AX (80312), GSK3α (4337), GSK3β (12456), cleaved-Caspase3 (9661), cleaved-PARP1 (5625), Chk1 (2360), p-Chk1 (2344), Snail (3879), Slug (9585), and RPA32 (2208) were from Cell Signaling Technology (Danvers, MA, USA). p-RPA32 (S4/S8; A300-245A) and p-RPA32 (S33; A300-246A) were from Bethyl Laboratories (Montgomery, Texas, USA). α-Tubulin (ab8035) and pericentrin (ab4448) were from Abcam (Cambridge, UK). Anti-GAPDH (AG019) antibody was from Beyotime (Shanghai, China).

### Cell lines

Human HCC1937, HCT-15, RKO, HCT-116, HT-29, UWB1.289, and UWB1.289 + BRCA1 cell lines were purchased from the American Type Culture Collection (Manassas, VA, USA). SW480 and SW620 cell lines were obtained from the Cell Bank of the Chinese Academy of Sciences Type Culture Collection (Shanghai, China). DR-U2OS and NHEJ–Hela cells were gifted by Ming Huang (Shanghai Institute of Materia Medica). Cells were cultured according to the supplier’s instructions and authenticated by short tandem repeat (STR) analysis performed by Genesky and tested for *Mycoplasma* contamination.

### Screening of drug combinations

BRCA1-deficient HCC1937 and BRCA2-deficient HCT-15 cells were used for screening the drug combinations. Prior to the screening, olaparib (OP) and niraparib (NP) were arrayed in 96-well plates and serially diluted 2-fold, and Sulforhodamine B (SRB) assay was used to analyze cytotoxicity to obtain a concentration that was 20% of the inhibition rate (IR). Data pertaining to single-agent activities of the drug library (99 agents targeting 50 classes of proteins) suggested that the active concentrations ranged from ~10 nM to ~10 μM. For the combination experiments, cells were treated with the compounds at three concentrations covering a 100-fold concentration range (10-fold dilution), with or without a fixed dose of OP or NP (~20% IR). In HCC1937 cells, 3.5 μM OP or 3.5 μM NP; in HCT-15 cells, 20 μM OP or 2.5 μM NP. A drug response score, indicating the effect of PARPi combined with the indicated agent, was calculated as ΔIR. For each screened drug dose, a ΔIR was calculated: ΔIR = inhibition rate of (combination IR3–indicated agent IR1–PARPi IR2). The color coding denotes the level of ΔIR (green [0% inhibition] to red [100% inhibition]).

### Cytotoxicity assays and combination analysis

Cells were treated with the indicated drug combinations and the IR on cell proliferation was determined using SRB assays as described previously^37^.

Combination Index (CI) was analyzed using the CompuSyn software with the Chou–Talalay equation^38^. CI < 1, CI = 1, and CI > 1 represented synergism, additive effect, and antagonism, respectively.

### Western blotting

The standard western blotting protocol was used to measure the cellular level of the indicated proteins, as described previously^18^.

### Generation of GSK3α and GSK3β KO cells using CRISPR/Cas9

Lentiviral transfection of cultured cells with pLentiCRISPRv2 vectors encoding GSK3α and GSK3β-specific CRISPR or control vectors (Obio Technology Co., Ltd., Shanghai, China) was performed according to the supplier’s instructions. The sequences of the oligonucleotide sgRNAs designed for GSK3α and GSK3β were 5′-ACCGGGCGCGGACTAGCTCGTTCG-3′ and 5′-ACCGGCCCAGAACCACCTCCTTTG-3′. The oligos were annealed and inserted into the lentiviral vector pLenti-U6-spgRNA v2.0-CMV-Puro-P2A-3Flag-spCas9. 293 T cells were transfected with 7.5 μg psPAX2, 2.5 μg pMD2.G, and 10 μg pLentiCRISPRv2 GSK3α/β sgRNA or pLentiCRISPRv2 vector. HCT-15 and RKO cells were then transduced with the lentiviruses. Finally, complete ablation of GSK3α or GSK3β expression was verified in the single-cell clones using western blotting.

### Flow cytometry

Cells were prepared for the analysis of cell cycle distribution or apoptosis as described previously^39^. Data were collected using a FACS Calibur Instrument (BD Biosciences, Franklin Lake, NJ, USA) and analyzed with the FlowJo software.

### Colony formation assay

Cells were plated in 6-well plates, cultured for 12 h, and then treated with various concentrations of drugs for another 7 days. After fixing, the colonies were stained with SRB and the optical density value was measured at 560 nm using a microplate reader (Molecular Devices, Sunnyvale, CA, USA).

### RNA interference

All small siRNAs were purchased from Genepharma (Shanghai, China). Transfection was conducted using Lipofectamine RNAiMAX (Invitrogen; Carlsbad, CA, USA) following the manufacture’s guidance. The sequences were as follows: negative control siRNA (siNC), 5′-UUCUCCGAACGUGUCACGUTT-3′; siGSK3β#1, 5′-GCUAGAUCACUGUAACAUATT-3′; siGSK3β#2, 5′- GAAAGCUAGAUCACUGUAATT-3′; siGSK3α#1, 5′-CCAGGACAAGAGGTTCAAGAA-3′; siGSK3α#2, 5′-CCUGGACAAAGGUGUUCAAAT-3′; siBRCA1, 5′-UCACAGUGUCCUUUAUGUA-3′; siSnai#1, 5′-GGACUUUGAUGAAGACCAU-3′; siSnai#2, 5′-GAUGCACAUCCGAAGCCAC-3′; siSlug#1, 5′-GGAGCAUACAGCCCUAUUA-3′; siSlug#2, 5′-GAUGCCCAGUCUAGGAAAU-3′.

### Transfection with GSK3β plasmids

Wild type (WT) or mutant GSK3β reconstituted cells were generated using lentiviral transfection of GSK3β KO1 HCT-15 cells with pLenti vectors encoding GSK3β WT or GSK3β Y216F cDNA (Obio Technology Co. Ltd.) followed by selection in presence of blasticidin.

The Flag-GSK3β WT plasmid was purchased from Obio Technology Co. Ltd. HCT-15 cells were transfected with the plasmid using lipofectamine 3000 (Invitrogen; Carlsbad, CA, USA) according to the manufacturer’s instructions.

### Immunofluorescence

Cells were prepared for immunofluorescence analysis as described previously^40^. Finally, the cells were stained with DAPI and imaged with a Leica immunofluorescence microscope (TCS-SP8 STED, Leica, Germany). The percentage of p-RPA32 (S33), γ-H2AX, RAD51 foci, and 53BP1 positive cells (≥5 foci/cell) was calculated based on the analysis of randomly chosen fields which included at least 50 cells. Mitotic spindle defects were examined by staining microtubules and centrosomes with anti-α-tubulin and anti-pericentrin antibody.

### HR and no—homologous end joining (NHEJ) repair assay

HR repair assays were performed as described previously using the DR-U2OS reporter cell line^41^. NHEJ repair assays were performed as described previously using the NHEJ–Hela reporter cell line^42^. Quantification was performed using 10,000 cells collected per sample. To examine the role of GSK3i or individual genes in DNA double-strand breaks (DSBs) repair, the cells were treated with the indicated agents or transfected with siRNA for 24 h. Then, the cells were transfected with a plasmid expressing I-SceI (pCBASce) for 48 h^43^. GFP-positive cells were quantified using flow cytometry.

### Quantitative real-time PCR

Total RNA was extracted using the HiPure Total RNA Mini Kit (Magen, Guangzhou, China) according to the manufacturer’s protocol. cDNA was generated using an RT reagent kit (TaKaRa, Tokyo, Japan). The quantitative real-time reverse transcription-polymerase chain reaction (qRT-PCR) reactions were performed using a 7500 Fast Real-time PCR System (Applied Biosystem, Grand Island, NY, USA). The primer sequences were as follows: 5′-ACCTTGGAACTGTGAGAACTCT-3′ (forward) and 5′-TCTTGATCTCCCACACTGCAATA-3′ (reverse) for BRCA1; 5′-GAGAAGGCTGGGGCTCATTT-3′ (forward) and 5′-AGTGATGGCATGGACTGTGG-3′ (reverse) for GAPDH. All experiments were performed in triplicate and normalized to the GAPDH transcript levels using the comparative CT method.

### In vivo anticancer activity experiments

Female nu/nu athymic BALB/cA mice (aged 5–6 weeks) were obtained from GemPharmatech (Jiangsu, China). All studies were conducted in compliance with the Institutional Animal Care and Use Committee guidelines of the Shanghai Institute of Materia Medica (Shanghai, China).

HCT-15, RKO, and HCT-15 KO xenografts were established by inoculating 5 × 10^6^ cells subcutaneously in the nude mice. When the xenografts reached a volume of 60–100 mm^3^, the mice were randomized into control and treatment groups as indicated. Simmiparib and LY2090314 alone or in a combination were injected every other day for the indicated period. Tumor growth was monitored by measuring the tumor size using calipers every other day and the tumor volume was calculated using the formula (length × width^2^)/2. Tumor tissues were collected 2 h after final dosing for immunoblotting or immunohistochemical staining. Images of immunohistochemical staining were captured using a NanoZoomer S210 (Hamamatsu, Japan) and processed using the NDP.scan.3.2.15 software.

### Statistical analyses

All data are presented as mean ± standard deviation (SD) or stand error of mean (SEM) from at least three independent experiments. The following methods were used to determine significance: unpaired *t* test, one-way ANOVA and two-way ANOVA. *p* < 0.05 was considered to be statistically significant. Normal distribution and variance were detected using the Shapiro–Wilk test and F test. All analyses were performed by the Prism.8 software (GraphPad, La Jolla, CA, USA).

## Results

### Drug combination screen identifies GSK3i as acting synergistically with PARPi

**Fig. 1:**
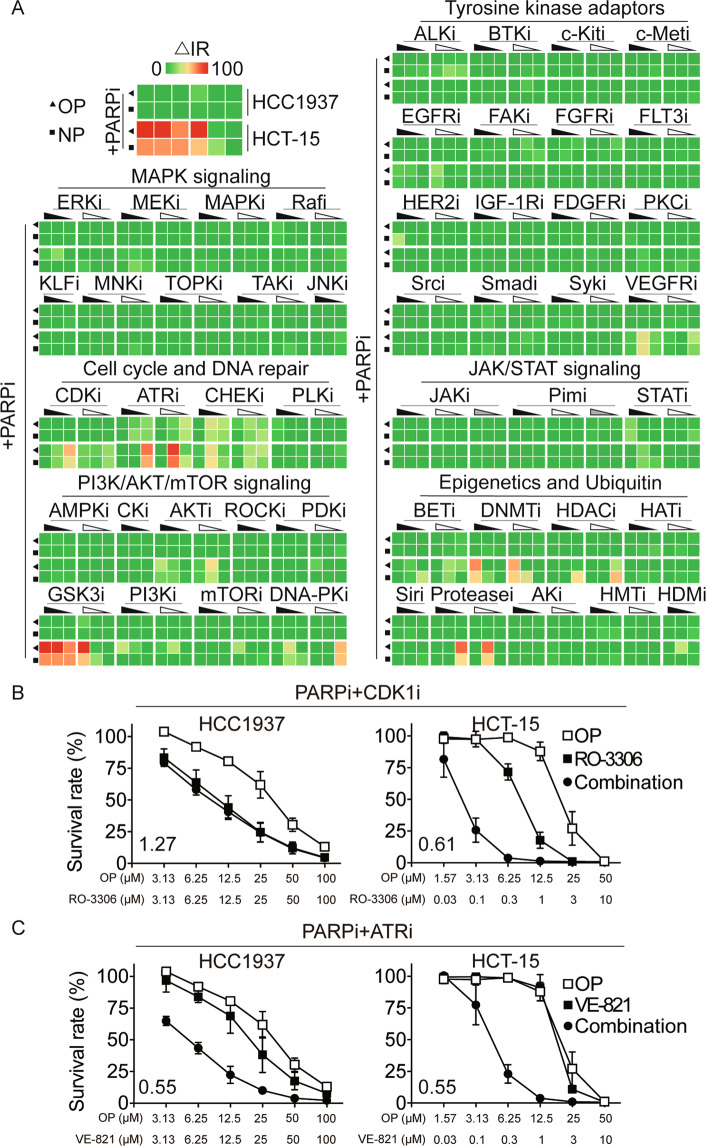
Drug combination screen identifies GSK3i as acting synergistically with PARPi. **A** Heatmap representation of the efficacy of drug combinations. Screening of drug combinations was performed in BRCA1-deficient HCC1937 and BRCA2-deficient HCT-15 cells. Prior to screening, olaparib (OP) and niraparib (NP) were arrayed in 96-well plates and serially diluted 2-fold, and Sulforhodamine B (SRB) assay was used to analyze cytotoxicity to obtain a concentration that was 20% of the inhibition rate (IR). Data pertaining to single-agent activities of the drug library (99 agents targeting 50 classes of proteins) suggested that the active concentrations ranged from ~10 nM to ~10 μM. For the combination experiments, cells were treated with the compounds at three concentrations covering a 100-fold concentration range (10-fold dilution), with or without a fixed dose of OP or NP (~20% IR). In HCC1937 cells, 3.5 μM OP or 3.5 μM NP; in HCT-15 cells, 20 μM OP or 2.5 μM NP. A drug response score, indicating the effect of PARPi combined with the indicated agent, was calculated as ΔIR. For each screened drug dose, a ΔIR was calculated: ΔIR = inhibition rate of (combination IR3–indicated agent IR1–PARPi IR2), the closed and open left angle triangle represent two different inhibitors from the same drug target, and their concentration decreases from left to right. **B** and **C** Effect of single agent and combination treatment on HCC1937 and HCT-15 cells viability for combinations of PARP inhibitor (olaparib, OP), plus CDK1 inhibitor (CDK1i) RO-3306 (**B**) or ATR inhibitor (ATRi) VE-821 (**C**). Cell viability was measured by Sulforhodamine B (SRB) assay. Combination index (CI) was calculated using CompuSyn software with the Chou–Talalay equation, and average CI values are presented (CI < 1, synergism; CI = 1, additive effect; CI > 1 antagonism). Data are from three independent experiments and expressed as mean ± standard deviation (SD).

To explore whether small-molecule inhibitors can sensitize cancer cells to PARPi, we performed a drug combination screen in BRCA1-deficient breast cancer cell line of HCC1937 and BRCA2-deficient CRC cell line of HCT-15, which express mutant-type BRCA1 or BRCA2 protein but modestly respond to PARPi. FDA-approved PARPi (olaparib and niraparib) and 99 well-characterized anticancer drugs targeting 50 classes of proteins belonging to indicate different kinds of the singling pathway were chosen for the initial screen (Table S1). Strikingly, a strong synergistic effect of GSK3i (CHIR99021 HCl and LY2090314) and PARPi (olaparib and niraparib) was observed in HCT-15 cells (Fig. 1A). Unsurprisingly, ATR inhibitors and CHEK1 inhibitors showed synergistic effects with PARPi (olaparib and niraparib) in HCC1937 and HCT-15 cells, which had been reported that both ATR and CHEK1 inhibitors increased the sensitivity to PARPi in a BRCA1-independent way^10,11^. Moreover, prior studies have demonstrated that inhibitors of BET, CDK1, HDAC, Protease, PI3K, and VEGFR could all decrease BRCA1 and other HRR factors at the protein level, thereby increasing the sensitivity of the cancer cell lines to PARP inhibition^44–49^. Consistent with the above conclusion, we found that these inhibitors displayed a synergistic effect with olaparib and niraparib in HCT-15 cells (Fig. 1A). As reported^50–53^, we also observed that olaparib and niraparib showed a synergistic effect in combination with inhibitors of DNMT, DNA-PK, mTOR, and HDM in HCT-15 cells (Fig. 1A).

To further confirm the accuracy of our screening results, we validated the above results in the combination of olaparib and CDK1 inhibitor (RO-3306) or ATR inhibitor (VE-821) using the CompuSyn model. Both combinations (i.e., olaparib + RO-3306 and olaparib + VE-821) caused obvious synergistic effects (CI < 0.7) in BRCA2-deficient HCT-15 cells, while only the olaparib and VE-821 combination produced synergistic effect (CI < 0.6) in the BRCA1-deficient HCC1937 cells (Fig. 1B, C). These data were consistent with the observation shown in Fig. 1A.

### GSK3 inhibition broadly sensitizes CRC cells to PARPi

**Fig. 2:**
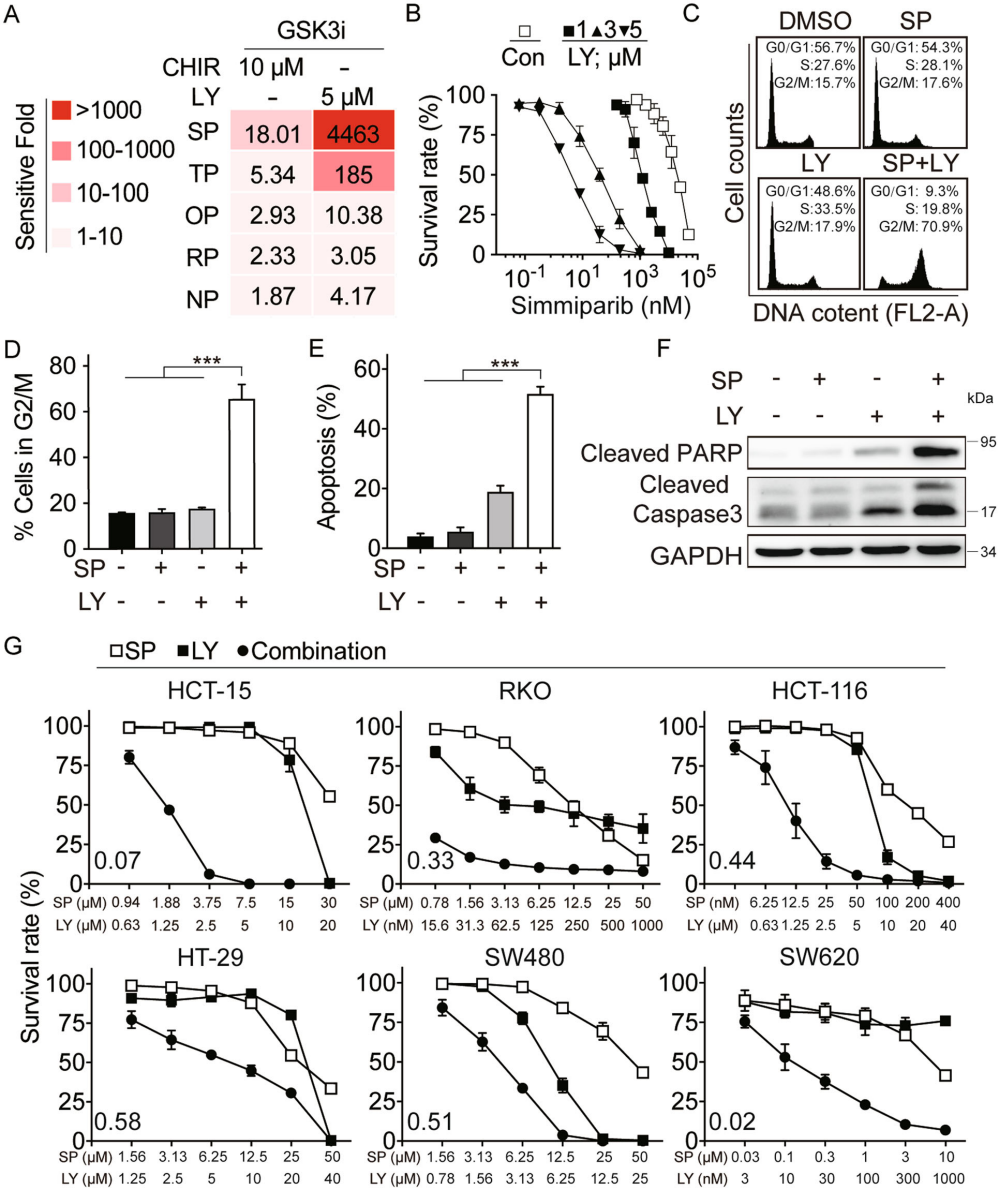
GSK3 inhibition sensitizes CRC cells to PARPi. **A** Change in sensitivity to PARPi when combined with GSK3i. HCT-15 cells were treated with various PARPi, including simmiparib, talazoparib, rucaparib, olaparib, and niraparib, without or with specific GSK3i CHIR99021 HCl (10 μM) or LY2090314 (5 μM) for 7 days. SP, simmiparib; TP, talazoparib; OP, olaparib; RP, rucaparib; NP, niraparib; CHIR, CHIR99021 HCl; and LY, LY2090314. Data (sensitive fold) are presented as the ratio of (IC_50_ of PARPi)/ (IC_50_ of PARPi plus GSK3i), indicating a reduction in IC_50_ of PARPi in the presence of GSK3i. Color intensity represents the value of sensitive fold. **B** Dose-response curves for HCT-15 cells treated with the indicated concentration of simmiparib (SP) with or without the LY2090314 (LY: 1, 3, and 5 μM) for 7 days. Data are from three independent experiments and expressed as mean ± SD. **C** and **D** G2/M arrest induced by single agent or the indicated combination in HCT-15 cells was determined using FACS. Cells were treated with 5 μM simmiparib (SP), 5 μM LY2090314 (LY), or a combination for 48 h and then subjected to FACS analysis. **C** Representative histograms are shown. **D** Percentage of cells in the G2/M phase expressed as mean ± SD from three independent experiments is shown. (***p < 0.001, one-way ANOVA). **E** Apoptosis induced by single agent or the indicated combination in HCT-15 cells. Cells were treated with 5 μM simmiparib (SP), 5 μM LY2090314 (LY), or a combination for 72 h and then analyzed using annexin V-FITC-PI-staining-based flow cytometry. Percentage of apoptotic cells expressed as mean ± SD from three independent experiments is shown. (***p < 0.001, one-way ANOVA). **F** Expression of apoptosis-related proteins in HCT-15 cells exposed to single agent or the indicated combination detected by western blotting. **G** GSK3 inhibitor, LY2090314 (LY), sensitized cells to PARP inhibitor, simmiparib (SP), in a panel of cells. The survival fraction and the average of CI values are shown from three independent experiments.

We next sought to validate the observed interactions between GSK3 activity and PARPi. To further investigate the effect of GSK3 activity on cellular response to PARPi, two specific GSK3i, LY2090314 (LY) and CHIR99021 HCl (CHIR), were used in combination with five PARPi, including olaparib, niraparib, rucaparib, talazoparib, and simmiparib. To exclude the synergistic effects that were simply due to cell cycle arrest, we chose the concentrations of GSK3i (CHIR ≤ 10 μM; LY ≤ 5 μM) that had no discernible effect on cell proliferation (Fig. S1A). Cells were treated with PARPi at eight concentrations, with or without LY2090314 or CHIR99021 HCl. The data showed that GSK3 inhibition strongly synergized with simmiparib (SP), talazoparib (TP), olaparib (OP), rucaparib (RP), and niraparib (NP) in HCT-15 cells (Fig. 2A). The synergistic effect decreased in the order of simmiparib (sensitive fold: up to ~4463-fold), talazoparib (~185-fold), olaparib (~10-fold), niraparib (~4-fold), and rucaparib (~3-fold) when combined with LY2090314. Thus, simmiparib, a potent and selective PARP inhibitor currently in phase I clinical trials in China, was the most strongly following GSK3 inhibition (No. CTR20160475). Moreover, the presence of GSK3i led to a decrease IC_50_ of simmiparib in a concentration-dependent manner in HCT-15 cells (Fig. 2B and Fig. S1B). In line with the synergistic effects between simmiparib and GSK3i, we observed enhanced G2/M arrest and apoptotic cell death induced by simmiparib when combined with LY2090314 (Fig. 2C–E) or CHIR99021 HCl (Fig. S1C–E). The protein levels of cleaved PARP1 (p85) and cleaved-Caspase 3 increased accordingly (Fig. 2F and Fig. S1F). The results indicated that simmiparib and GSK3i combination treatment significantly suppressed tumor cell growth, caused cells to accumulate in G2/M of the cell cycle, and induced remarkably apoptotic response.

To determine whether these synergies extend across other tumor cells, we used additional BRCA-proficient CRC cell lines (RKO, HCT-116, SW480, SW620, and HT-29) to detect the synergistic effect of PARPi and GSK3i. The data showed that GSK3 inhibition strongly synergized with simmiparib in all the BRCA-proficient CRC cells (CI < 0.6), as well as HCT-15 cells (Fig. 2G and Fig. S1G). Consistently, no combination activity (CI > 1) was observed in BRCA1-deficient HCC1937 cell lines (Fig. S1H). This finding suggested a broader benefit of PARPi combined with GSK3i in BRCA2-deficient and BRCA-proficient CRC cells.

### GSK3β depletion selectively sensitizes cancer cells to PARPi, topoisomerase (Top) I inhibitor, and hydroxyurea

**Fig. 3:**
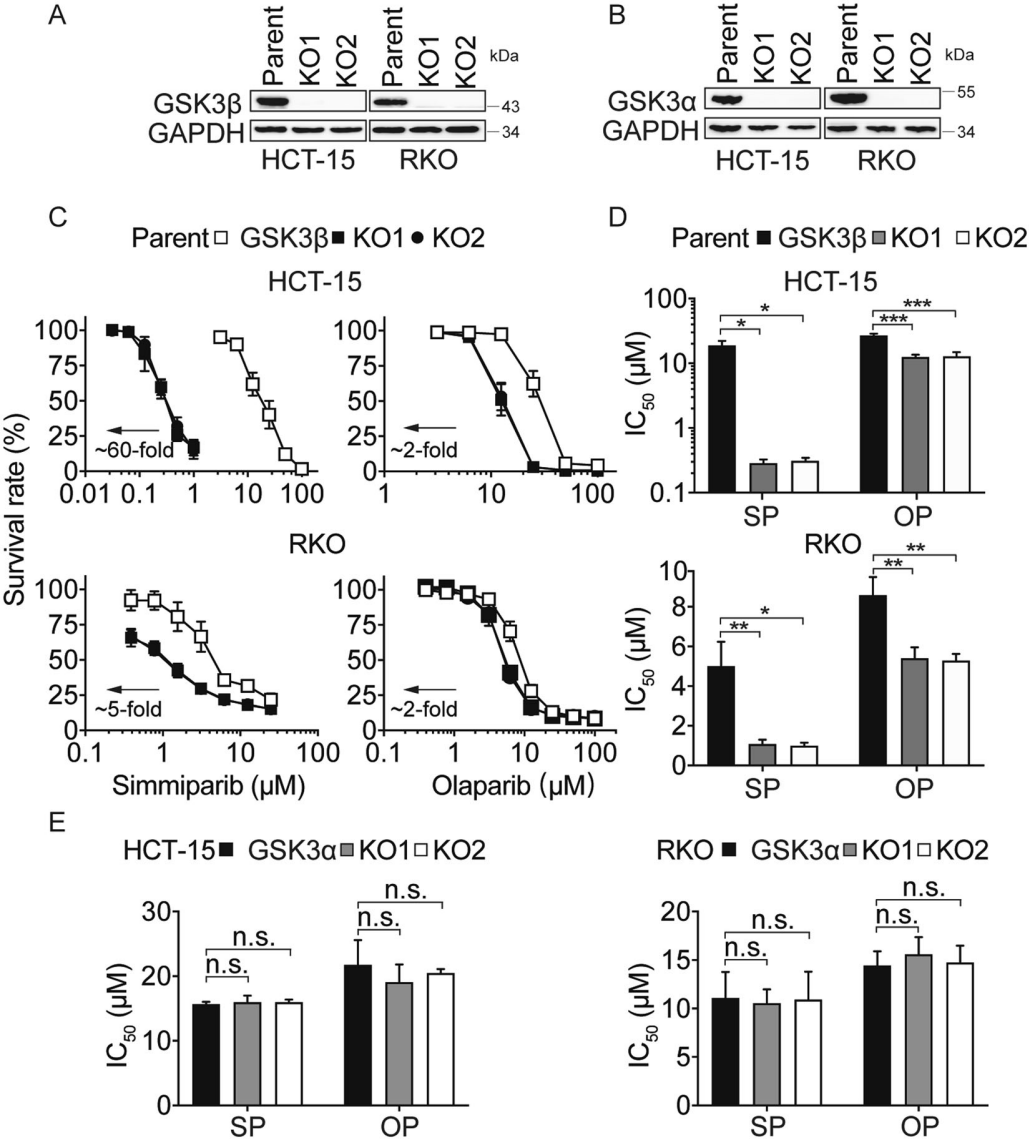
GSK3β depletion selectively sensitizes cancer cells to PARPi. **A** and **B** Levels of GSK3β and GSK3α protein in different GSK3β^−/−^ or GSK3α^−/−^ clonal variants (KO1 and KO2) of HCT-15 and RKO (parent) cells were detected using western blotting. **C**–**E** The change in sensitivity to PARPi following GSK3β (**C** and **D**) or GSK3α depletion (**E**). Cells were treated with simmiparib (SP) and olaparib (OP) for 7 days then subjected to SRB assays. The IC_50_ values are expressed as mean ± SD from three independent experiments. (*p < 0.05, **p < 0.01, ***p < 0.001, n.s. not significant, *t* test).

There are two highly homologous forms of GSK3 in humans, GSK3α and GSK3β, that have different tissue-specific functions and substrates^19,20^. As GSK3i (LY2090314 and CHIR99021 HCl) block both GSK3α and GSK3β activity, we next generated *GSK3α* null and *GSK3β* null cells lines using CRISPR/Cas9 technique in HCT-15 and RKO cells, respectively (Fig. 3A, B). Relative to the parental cells, the *GSK3β* KO cells (KO1 and KO2) displayed up to 60-fold increased sensitivity to the PARPi, simmiparib (Fig. 3C, D). However, GSK3α depletion did not affect the cellular sensitivity to PARPi (Fig. 3E). These results indicated that depletion of GSK3β selectively sensitized cancer cells to PARPi.

To investigate the possible involvement of GSK3β in sustaining genomic stability, we examined whether GSK3i, LY2090314, synergized with different DNA-damaging agents known to generate different forms of DNA lesions in HCT-15 cell line. The results revealed that GSK3i synergized with irinotecan (CPT-11, Top I inhibitor; CI < 0.4) and hydroxyurea (HU, DNA synthesis inhibitor; CI < 0.5), but not adriamycin (ADR, Top II inhibitor; CI > 1) or etoposide (VP-16, Top II inhibitor; CI > 1) (Fig. S2A–C). Similarly, GSK3β depletion, but not GSK3α, significantly increased the cellular sensitivity to CPT-11 and HU (Fig. S2D, E).

### GSK3β is required for the HRR of DSBs

**Fig. 4:**
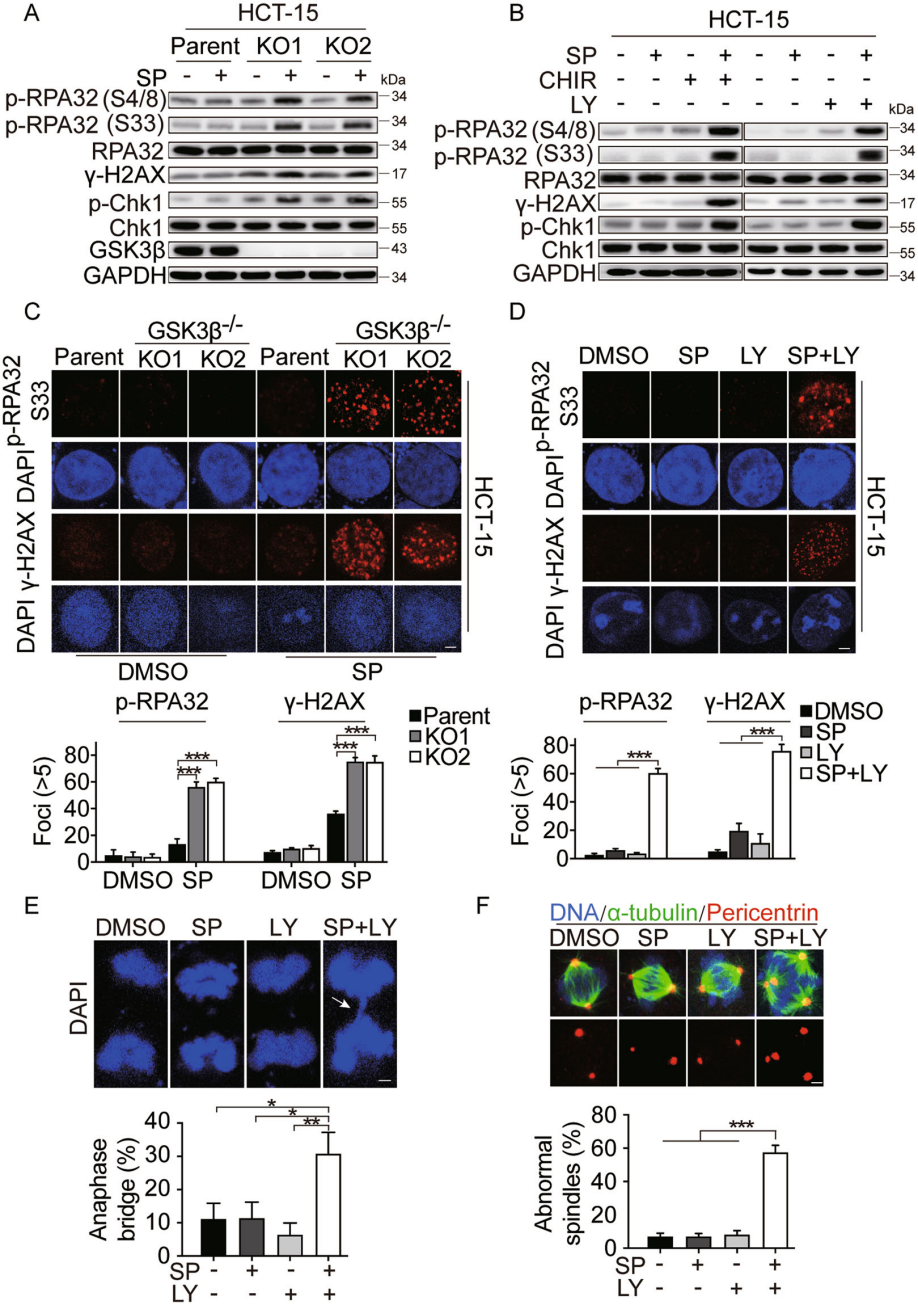
Combined GSK3β and PARP inhibition increases DNA damage and mitotic aberrancies. **A** Western blot analysis of indicated proteins in HCT-15 cells (parent) or corresponding GSK3β-depleted single clone (KO1 and KO2) cells treated with 5 μM simmiparib (SP) for 48 h. **B** Western blot analysis of indicates proteins in HCT-15 cells treated with 5 μM simmiparib, GSK3i (10 μM CHIR99021 HCl or 5 μM LY2090314), or a combination for 48 h. SP, simmiparib; CHIR, CHIR99021 HCl; LY, LY2090314. **C** and **D** Representative images of p-RPA32 (S33) and γ-H2AX foci in HCT-15 (parent) and their GSK3β-depleted single clone (KO1 and KO2) cells treated with 5 μM simmiparib (**C**) or HCT-15 cells following treatment with 5 μM simmiparib, GSK3i (5 μM LY2090314), or a combination for 48 h (**D**). Nuclei were stained with DAPI. Scale bar: 2 μm. Cells that contained five or more p-RPA32 or γ-H2AX foci/nucleus were considered as positive cells. At least 50 cells were analyzed for each experiment and condition. All data are expressed as mean ± SD from three independent experiments. (**C**: ***p < 0.001, *t* test; **D**: ***p < 0.001, one-way ANOVA). SP, simmiparib; CHIR, CHIR99021 HCl; LY, LY2090314. **E** The combination of GSK3i and PARPi increases anaphase bridge-positive cells. HCT-15 cells were treated with 5 μM simmiparib (SP), 5 μM LY2090314 (LY), or a combination for 48 h. Cells were examined by DAPI-staining and microscopy for chromatin bridges. Representative images (upper panel; scale bar: 2 μm) and percentages of anaphase bridge-positive cells are shown (lower panel; *p < 0.05, **p < 0.01, one-way ANOVA). Anaphase cells (Control: 45 cells; SP: 44 cells; LY: 47 cells; SP + LY: 52 cells) from five independent experiments. Data are expressed as mean ± stand error of mean (SEM). **F** The combination of GSK3i and PARPi impairs mitotic spindles. HCT-15 cells were immunostained with α-tubulin (green) for mitotic spindles and pericentrin (red) for centrosomes after treated with 5 μM simmiparib (SP), 5 μM LY2090314 (LY), or a combination for 48 h. Nuclei were stained with DAPI. Representative images (upper panel; scale bar: 2 μm) and percentages of abnormal spindles are shown (lower panel; ***p < 0.001, one-way ANOVA). At least 50 cells were analyzed for each experiment and condition. Data are expressed as mean ± SD from three independent experiments.

**Fig. 5:**
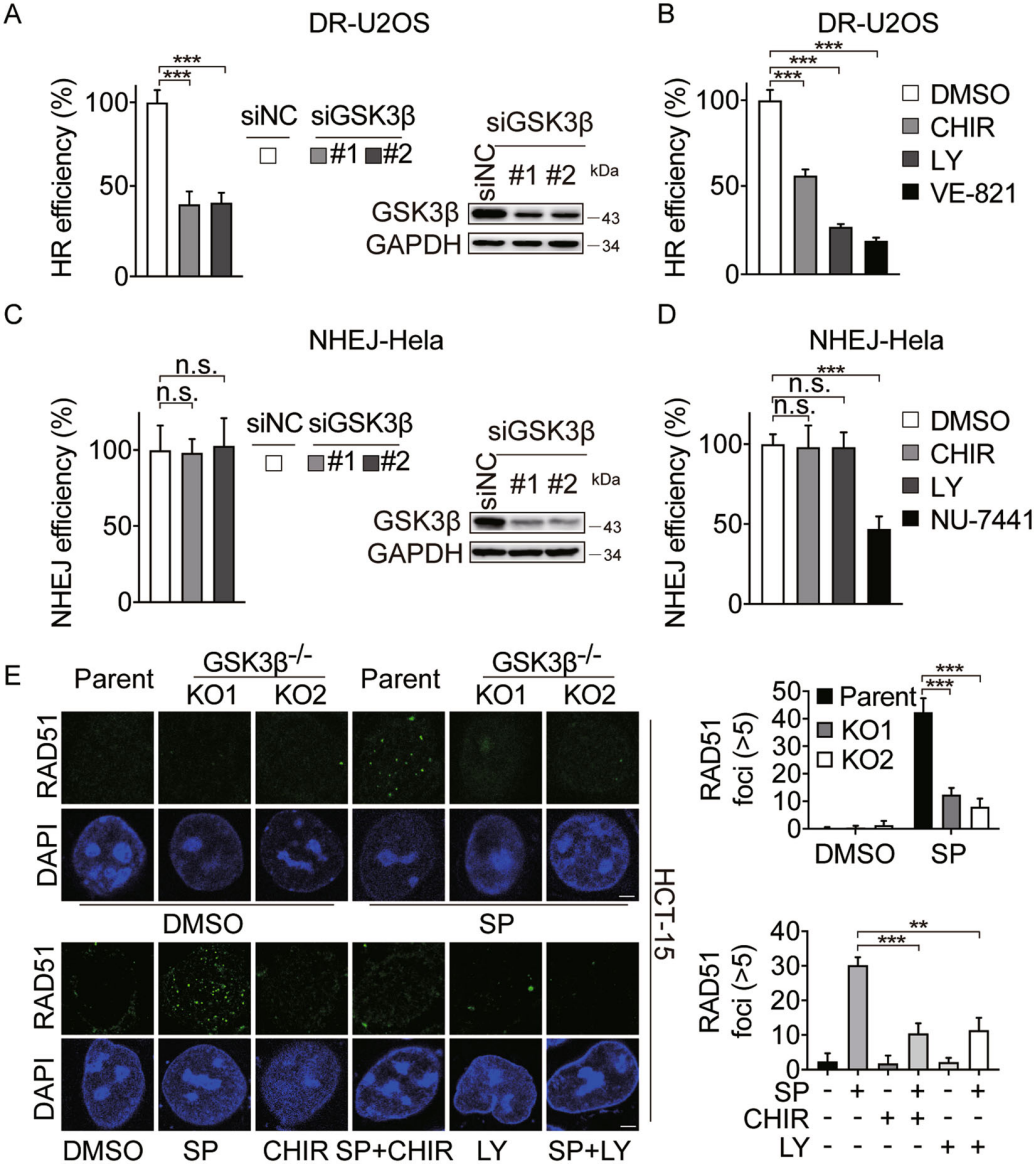
GSK3β is required for the homologous recombination repair of DSBs. **A–D** DR-U2OS (**A** and **B**) or NHEJ–Hela (**C** and **D**) cells were treated with siRNA, GSK3β inhibition (5 μM CHIR99021 HCl or 5 μM LY2090314), or positive control of VE-821 (5 μM, in HR assays) or NU-7441 (10 μM, in NHEJ assays) for 24 h, followed by I-SceI transfection. GFP-positive cells were analyzed by flow cytometry 48 h later. Data are expressed as mean ± SD from three independent experiments. (***p < 0.001, n.s. not significant, *t* test). CHIR, CHIR99021 HCl; LY, LY2090314. **E** Representative images of RAD51 foci in HCT-15 (parent) and their GSK3β-depleted single clone (KO1 and KO2) cells treated with 5 μM simmiparib (Upper) or HCT-15 cells following treatment with 5 μM simmiparib, GSK3i (10 μM CHIR99021 HCl or 5 μM LY2090314), or a combination for 48 h (Lower). Nuclei were stained with DAPI. Scale bar: 2 μm. Cells that contained five or more RAD51 foci/nucleus were considered as RAD51-positive cells. At least 50 cells were analyzed for each experiment and condition. All data are expressed as mean ± SD from three independent experiments. (**p < 0.01, ***p < 0.001, *t* test). SP, simmiparib; CHIR, CHIR99021 HCl; LY, LY2090314.

Although PARPi, Top I inhibitor, and HU cause different forms of DNA lesions, these agents are known to selectively kill proliferating cancer cells by causing replication-dependent DSBs^54–56^. For this reason, we compared the occurrence of drug-induced DSBs in *GSK3β* KO and parental cells, using γ-H2AX as a marker. Upon simmiparib treatment, a higher level of γ-H2AX protein accumulated in *GSK3β* KO cells compared to the parental cells (Fig. 4A and Fig. S3A). In response to replication stress, RPA32 (p-S33 and p-S4/8) and Chk1 are primarily phosphorylated by ATR or DNA-PK, which are widely used as the markers of replication stress^57^. The data showed that no changes of p-RPA32 and p-Chk1 protein levels were observed in *GSK3β* KO cells. However, simmiparib caused a dramatic increase of p-RPA32 (S33 and S4/8) and p-Chk1 protein levels in *GSK3β*-depleted but not in parental cells (Fig. 4A). These results were further supported by the enhanced p-RPA32, p-Chk1, and γ-H2AX protein levels in cells treated with a combination of PARPi and GSK3i (Fig. 4B and Fig. S3B); and the observation was recapitulated using an immunofluorescence assay to stain nuclear p-RPA32 (S33) and γ-H2AX foci (Fig. 4C, D and Fig. S3C). However, the level of DSBs has similarly induced in *GSK3α* null cells and parental cells (Fig. S3D). Replication stress may stall chromosome duplication, promoting premature mitotic entry in the presence of DNA lesions, and resulting in increased anaphase bridge formation. Indeed, the combination of GSK3i and PARPi led to a significantly increased number of cells contain anaphase bridges (Fig. 4E). Similarly, multipolar and distorted spindles occurred at a higher frequency in cells cotreated with GSK3 and PARP inhibitors (Fig. 4F). Taken together, these data showed that combined PARP and GSK3β inhibition increased replication-dependent DSBs and the percentage of mitotic aberrancy. Replication-dependent DSBs lesions are known to be predominantly repaired by HR, a repair process requiring homologous DNA sequence as a template. To test whether GSK3β inhibition and knockdown cells were defective in HRR, we chose a well-characterized reporter assay using the DR-U2OS, a human osteosarcoma cell line with chromosomally integrated HR reporter gene containing an I-SceI recognition sequence^41^. In this cell line, HRR using a direct repeat within the reporter cassette as a template results in an intact GFP gene, which can be detected by flow cytometry. The data showed that GSK3β knockdown using two independent siRNAs remarkably decreased the HR efficiency triggered by I-SceI (Fig. 5a). Consistently, the GSK3i, CHIR99021 HCl, and LY2090314, significantly reduced the capacity of HRR, in which ATR inhibitor, VE-821, was used as a positive control (Fig. 5B). In contrast, depletion or inhibition of GSK3β had no impact on non-homologous end joining (NHEJ) repair, as measured by a similar NHEJ reporter system using NHEJ–Hela cells. In this assay, DNA-PK inhibitor, NU-7441, was used as a positive control (Fig. 5C, D)^42^. However, GSK3α silencing had no impact on HR or NHEJ efficiency (Fig. S4A, B). Additionally, we observed impaired RAD51 foci formation in *GSK3β* KO cells or GSK3i-treated cells which further strengthened the deficiency in HRR (Fig. 5E and Fig. S4C). The combination of GSK3i and PARPi had no additional effect on 53BP1 foci formation compared to PARPi alone (Fig. S4D). Together, these data identified a previously unappreciated role of GSK3β in HRR, which echoed our findings that GSK3β inhibition and depletion affected cell sensitivity to PARPi, Top I inhibitor, and HU.

### GSK3β depletion represses the expression of BRCA1

**Fig. 6:**
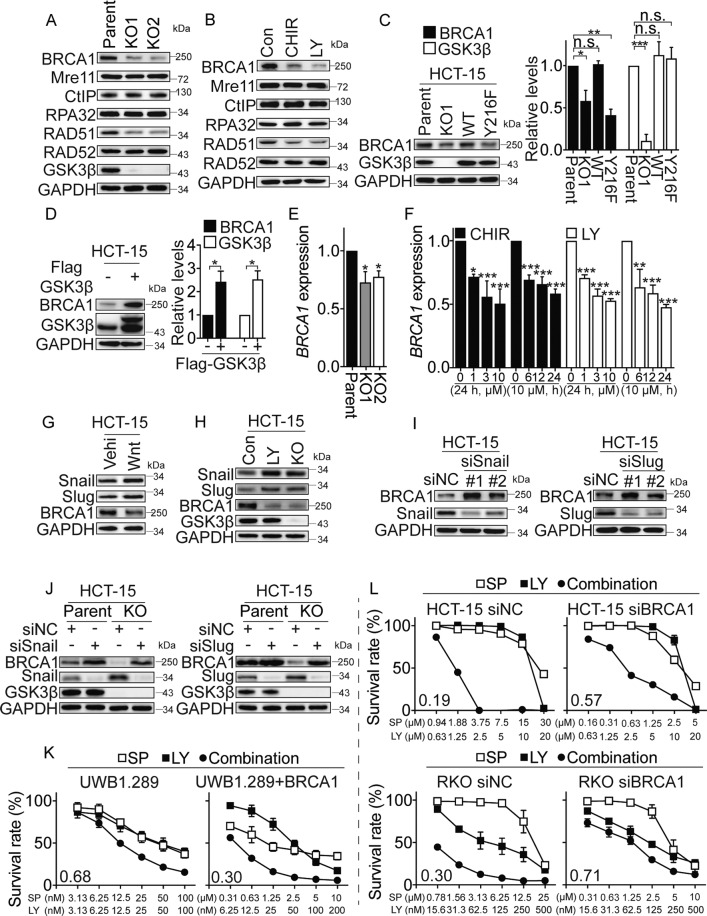
GSK3β depletion represses the expression of BRCA1. **A** Levels of DNA repair-related proteins in the parental HCT-15 and their GSK3β-depleted single clone (KO1 and KO2) cells determined by western blotting. **B** Levels of DNA repair-related proteins in HCT-15 cells following treatment with 10 μM CHIR99021 HCl (CHIR) or 5 μM LY2090314 (LY) determined for 48 h by western blotting. **C** BRCA1 protein level was partially restored in HCT-15 GSK3β-depleted cells transfected with full-length WT-*GSK3β* cDNA (WT) but not with mutated*-GSK3β* cDNA (Y216F). The relative intensities of the bands were quantified by Image J software and normalized to GAPDH levels. All data are expressed as mean ± SD from three independent experiments. (*p < 0.05, **p < 0.01, ***p < 0.001, *t* test). **D** Exogenous expression of GSK3β in HCT-15 cells increased BRCA1 protein level. The relative intensities of the bands were quantified by Image J software and normalized to GAPDH levels. All data are expressed as mean ± SD from three independent experiments. (*p < 0.05, *t* test). **E** mRNA expression of *BRCA1* in the parental HCT-15 and their GSK3β-depleted single clone (KO1 and KO2) cells was detected by qRT-PCR. (*n* = 3, *p < 0.05, *t* test). **F** *BRCA1* mRNA levels in HCT-15 cells treated with GSK3i, CHIR99021 HCl (CHIR), and LY2090314 (LY), for indicated times and concentrations. (*n* = 3, *p < 0.05, **p < 0.01, ***p < 0.001, one-way ANOVA). **G** and **H** The Snail and Slug expression levels were negatively correlated with the levels of BRCA1. The expression of indicated proteins was analyzed by western blotting in the HCT-15 cells transfected with the full-length *Wnt3a* cDNA (**G**), or treated with GSK3i LY2090314 (LY) and depleted GSK3β (KO; **H**). **I** Knockdown of Snail and Slug increased BRCA1 expression in HCT-15 cells. Cells were treated with siRNAs targeting human Snail, Slug, or siNC for 48 h. The expression of indicated proteins was analyzed by western blotting. **J** Silencing of Snail and Slug restored the levels of BRCA1 in GSK3β KO cells. HCT-15 and GSK3β KO cells were treated with siSnail, siSlug, or siNC for 48 h. **K** and **L** Effect of single agent and combination treatment on indicated cells viability for combinations of PARP inhibitor, simmiparib (SP), plus GSK3 inhibitor LY2090314 (LY). UWB1.289 (carrying a BRCA1 mutation) and UWB1.289 + BRCA1 (restored with wild-type BRCA1) cells were analyzed 7 days after treatment with simmiparib (SP), LY2090314 (LY), or a combination (**K**). HCT-15 and RKO cells were transfected with siBRCA or siNC for 24 h and then followed by treatment of simmiparib (SP), LY2090314 (LY), or a combination for 7 days (**L**). Cell viability was measured by SRB assay. Combination index (CI) was calculated using CompuSyn software with the Chou–Talalay equation, and average CI values are presented (CI < 1, synergism; CI = 1, additive effect; CI > 1 antagonism). Data are from three independent experiments and expressed as mean ± SD.

To understand how GSK3β is involved in HRR, we analyzed the protein levels of the key factors involved in the HR pathways using western blotting. *GSK3β* KO cells showed a marked reduction in BRCA1 protein levels, whereas the levels of Mre11, CtIP, RPA32, and RAD52 were not affected (Fig. 6A and Fig. S5A). Similarly, inactivation of GSK3β by CHIR99021 HCl and LY2090314 treatment led to a marked decrease in BRCA1 protein level in a concentration- and time-dependent manner (Fig. 6B; Fig. S5B–D). Furthermore, we found that GSK3β depletion and inhibition reduced RAD51 protein level in HCT-15 cells but not in RKO cells (Fig. 6A, B and Fig. S5A, B). Therefore, we assessed the effect of LY2090314 on BRCA1 and RAD51 protein levels in other CRC cells (HCT-116, HT-29, SW480, and SW620). LY2090314 modestly decreased RAD51 protein level in HT-29 cells, while it consistently decreased BRCA1 protein levels in all the lines assessed (Fig. S5E). We thus focused on BRCA1 as a likely mediator of the GSK3i effect. We transfected *WT*-*GSK3β* (WT) or a kinase-inactive mutant *GSK3β*^Y216F^ (Y216F) cDNA into HCT-15 KO cells, and obtained the corresponding variants that expressed the WT or Y216F GSK3β proteins. As expected, reconstitution with WT-GSK3β, but not Y216F-GSK3β, partially restored the BRCA1 protein level (Fig. 6C), suggesting that GSK3β enzymatic activity was required to retain protein. Ectopically expressed Flag-GSK3β also resulted in an increase in BRCA1 protein in the parental HCT-15 cells, which further suggested a strong association between GSK3β and BRCA1 (Fig. 6D).

The reduction in BRCA1 protein level appeared to be a result of transcriptional repression, as RT-PCR revealed that the *GSK3β* KO cells had reduced *BRCA1* mRNA expression (Fig. 6E and Fig. S5F). In addition, cells treated with GSK3i (CHIR99021 HCl and LY2090314) showed a reduced mRNA expression of *BRCA1* in a time- and concentration-dependent manner (Fig. 6F and S5G). However, BRCA1 protein levels were not affected by MG-132 treatment in *GSK3β* KO or GSK3i-treated cells (Fig. S5H). Collectively, these data implied that GSK3β may repress BRCA1 transcription and protein expression in an enzyme-dependent manner. It remained unclear how GSK3β affected the mRNA expression of *BRCA1*. Previous studies have reported that Wnt3a–GSK3β signaling pathway regulated EMT and BRCA1 expression through stabilizing Slug and Snail in breast cancer cells^58^. We hence speculated that GSK3β inhibition and depletion might suppress BRCA1 expression by activating Wnt3a/Slug/Snail pathway in our system. Indeed, BRCA1 protein levels were suppressed when the levels of Slug or Snail were increased by overexpression of Wnt3a (Fig. 6G), or inactivating GSK3β (Fig. 6H) in HCT-15 cells. Conversely, BRCA1 protein levels were increased when either Slug or Snail expression was silenced in HCT-15 cells (Fig. 6I). Furthermore, depletion of Snail and Slug nearly restored BRCA1 levels in GSK3β-deficient cells (Fig. 6J), suggesting Snail and Slug are key components of GSK3β signaling that regulates BRCA1 expression. Together, these results suggested that Snail and Slug expression levels were negatively correlated with the levels of BRCA1, supporting the conclusion that Wnt3a/GSK3β/Slug/Snail axis regulated BRCA1 expression in HCT-15 cells.

Our data showed that depletion of GSK3β resulted in impaired HR and reduced BRCA1 expression in vitro. Moreover, combined GSK3 and PARP inhibition has yielded encouraging results in BRCA2 deficient or HR proficient colon cancer cells but not on BRCA1 deficient breast cancer HCC1937 cells. To further evaluate whether BRCA1 is important for this combination, we examined the effects of GSK3i (LY or CHIR) or PARPi SP alone or their combination on cell viability in a pair of ovarian cancer UWB1.289 (carrying a *BRCA1* mutation, BRCA1-null), and UWB1.289 + BRCA1 cells, in which wild-type BRCA1 was restored. As expected, the average CI values were significant decreased in UWB1.289 + BRCA1 cells (Average CI = 0.38 and 0.30) compared to UWB1.289 cells (Average CI = 0.87 and 0.68) (Fig. 6K and Fig. S5I). Furthermore, the synergistic effect was significant reduced in the HCT-15 and RKO cells upon BRCA1 knockdown (Fig. 6L; Fig. S5J, K). These data suggested that BRCA1 maybe play a certain role in this new combination strategy.

### PARPi and GSK3β inhibition are synergistic in vivo

**Fig. 7:**
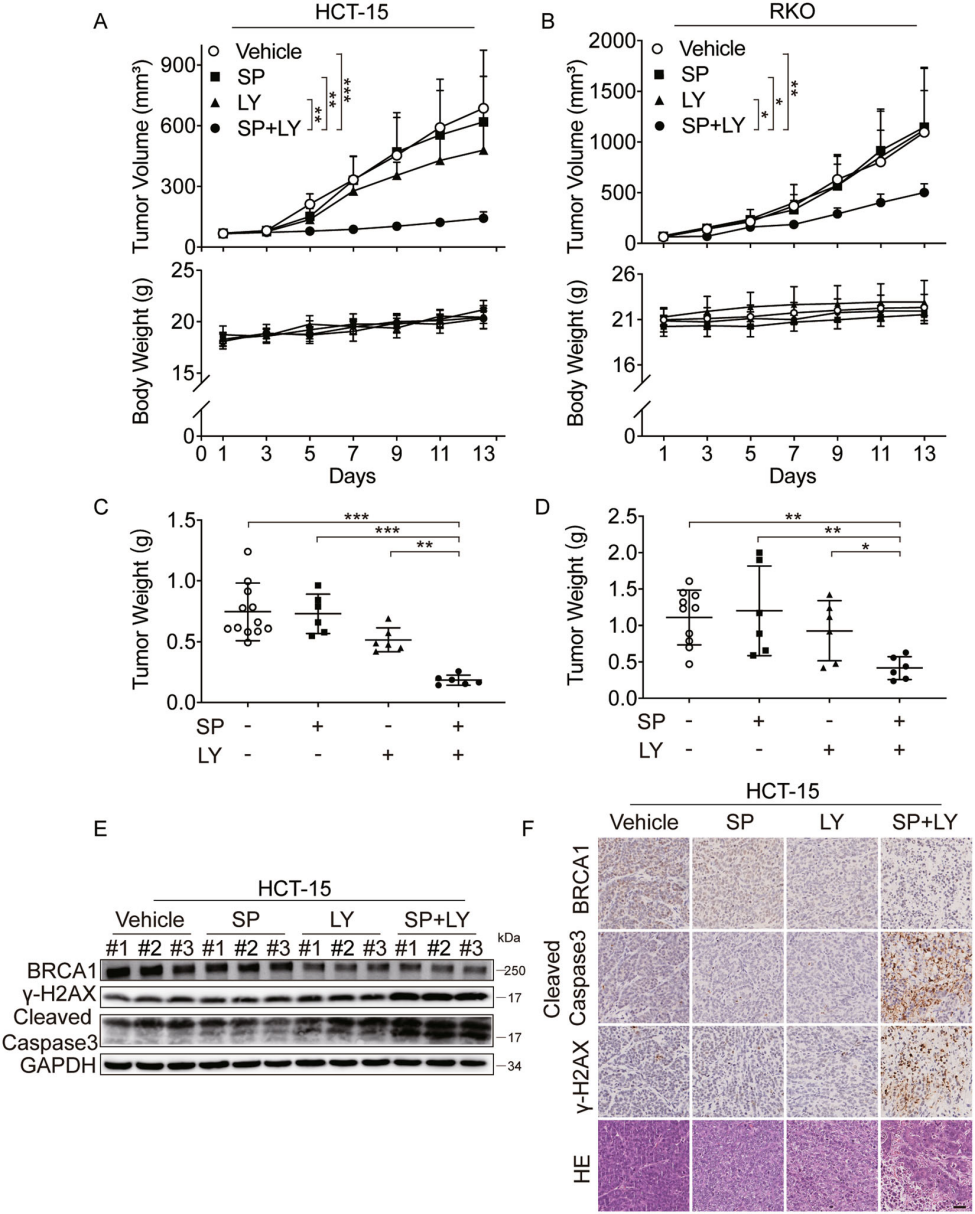
PARP and GSK3β inhibition are synergistic in vivo. **A**–**D** Mice bearing subcutaneous xenografts were treated with simmiparib (SP, *i.v*.) or LY2090314 (LY, *i.v*.) every other day, either alone or in a combination. Tumor volume, body weight, and tumor weight were separately plotted. **A** and **C** show the effect of simmiparib (SP, 50 mg/kg) and LY2090314 (LY, 50 mg/kg), alone or in a combination on BRCA2-deficient HCT-15 xenografts; **B** and **D** show the effect of simmiparib (SP, 30 mg/kg) and LY2090314 (LY, 30 mg/kg), alone or in a combination on BRCA-proficient RKO xenografts. **E** and **F** Levels of BRCA1, γ-H2AX, and cleaved-Caspase3 in HCT-15 xenografts determined by western blotting (**E**) or immunohistochemistry (**F**) analysis. Scale bar, 20 μm. (*n* = 6, *p < 0.05, **p < 0.01, ***p < 0.001, two-way ANOVA).

Our data thus far indicated that GSK3 inhibition strongly synergized with PARPi in BRCA2-deficient and BRCA1/2-proficient cancer cells in vitro. We further validated this therapeutic potential using xenograft mice models. BRCA2-deficient HCT-15 cells and BRCA-proficient RKO cells were subcutaneously injected into nude mice, and once tumor volume reached ~70 mm^3^, either simmiparib or LY2090314, alone or in combination, was injected every other day for 14 days. Notably, the combination of these two agents significantly inhibited the growth of the tumor in the HCT-15 and RKO xenograft mouse model, although the tumor growth in the single-agent groups was not affected following simmiparib or LY2090314 treatment (Fig. 7A, B). Consistently, the tumor burden was significantly reduced as measured by the weight of dissected tumors (Fig. 7C, D). The increased response to the combination treatment was associated with an increased number of DSBs lesions (as indicated by γ-H2AX levels), as well as increased the levels of cleaved-Caspase3 in the combined treatment group (Fig. 7E, F). In support of the mechanism identified in this study, the GSK3i group showed decreased BRCA1 protein level (Fig. 7E, F). All the tested compounds caused no obvious loss of weight of the nude mice (Fig. 7A, B) and were well tolerated during the drug administration.

To further validate the impact of GSK3β on in vivo sensitivity to PARPi, we used the HCT-15 *GSK3β* KO cells and parental cells to establish xenograft models in nude mice. As expected, administration of simmiparib significantly inhibited the growth of *GSK3β* KO tumor xenografts, but not the parental tumor xenografts. (Fig. S6A, B). Consistently, there was a significant decrease in BRCA1 protein level and increase in γ-H2AX level in the *GSK3β* KO tumor xenografts treated with simmiparib (Fig. S6C). These data demonstrated that inhibition or depletion of GSK3β could enhance the in vivo sensitivity to simmiparib without toxicity.

## Discussion

To identify effective drug combinations for *BRCA*-mutated cancer cells with de novo PARPi resistance, we tested the cellular effect of a panel of compounds either alone or in combination with PARPi in *BRCA1*-mutated HCC1937 and *BRCA2*-mutated HCT-15 cells. Through this in vitro screen, we identified that a quarter of the oncological drugs and kinase inhibitors tested displayed synergy with PARPi in HCT-15 cells. These agents have included inhibitors of the DNA damage and cell cycle checkpoint (targeting ATR, CHK1, or CDK1), PI3K pathway (targeting PI3K, AKT, or mTOR), and epigenetics regulators (targeting DNMT, HDAC, and BET), and VEGFR. More importantly, the data suggested that GSK3 inhibition was most effective in enhancing the efficacy of PARPi. In conclusion, based on comprehensive and systematic screening of compounds, this study identified compounds that are capable of synergizing with PARPi.

Some of the synergistic interactions described in our screening were identified in previous studies^10,11,44–46,48–50,52^. For example, PARP inhibition was shown to synergize with: (1) PI3K pathway antagonism in BRCA-proficient triple-negative breast cancer cells, (2) ATR–Chk1 inhibition in PARPi-resistant BRCA-deficient cancer cells and high-grade serous ovarian cancer cells, (3) BET inhibition in multiple tumor lineages, (4) VEGFR antagonism in ovarian cancer cells. Our screens also revealed that the synergistic effect between PARPi and these compounds was far more prevalent in BRCA2-deficient HCT-15 cells (~25%) than in BRCA1-deficient HCC1937 cells (~4%), which implicated that populations with *BRCA1* or *BRCA2* mutations may benefit differently from PARPi-based combination therapies.

In this study, a strong synergistic effect between GSK3i and PARPi was observed in multiple CRC cell lines with diverse genetic backgrounds. Further in vivo studies showed that this new combination markedly suppressed tumor growth of HCT-15 and RKO tumor xenografts, without additional toxicity. Previous studies have demonstrated that olaparib combined with irinotecan displayed high toxicity concerns and no antitumor efficacy in CRC patients^59^. In this study, our results suggested that the combination of GSK3i and PARPi may produce encouraging responses with optimum tolerance in CRC patients. Intriguingly, simmiparib showed better synergy compared with other PARPi when combined with GSK3i (Fig. 2A). Moreover, GSK3β depletion resulted in superior sensitivity (60-fold) to simmiparib compared to olaparib (only 2-fold) (shown in Fig. 3B). A recent study demonstrated that simmiparib displayed more potent cytotoxicity (>43-fold, in vitro; >10-fold, in vivo) than olaparib, but showed no significant difference in PARP1–DNA trapping^9,18^. It is thus conceivable that the efficacy of PARPi in combination with GSK3 inhibition is more tightly correlated with PARPi cytotoxicity than PARP1-DNA trapping. Similarly, LY2090314 has more potent activity against the GSK3α/β than CHIR99021 HCl, and the former displayed better synergy with simmiparib compared with the latter (Fig. 2A).

In addition to regulating cellular processes including metabolism, growth, and survival, GSK3β also mediates the repair of DNA DSBs through phosphorylation of p53 binding protein 1 (53BP1)^60^ and modulates the HRR pathway by phosphorylating the Fanconi anemia-associated protein (FAAP2), an important component of the Fanconi anemia complex involved in the repair of DNA inter-strand cross-links^61^. Furthermore, GSK3i altered the level of proteins involved in DNA repair, such as ATR-interacting protein (ATRIP), topoisomerase IIβ-binding protein (TopBP1)^34^, tumor protein p53-induced nuclear protein 1 (TP53INP1)^35^, and Tap63^62^. In addition, GSK3β inhibition has been shown to enhance ionizing radiation-based sensitivity in vitro^63^ and in xenograft models^60^. Previous studies demonstrated that inhibition of GSK3β accelerated NHEJ-mediated DSB repair through regulating TRAX in neurons^64^. However, it was also reported that inhibition of GSK3 enhanced NHEJ mediated DNA repair exclusively in normal cells but not cancer^65^. This was consistent with our study showing that GSK3α/β depletion did not affect the efficiency of NHEJ in cancer cells (Fig. 5 and Fig. S4). Meanwhile, our results advanced the current understanding of the role of GSK3β by showing that GSK3β is essential for DSBs in HRR by affecting BRCA1 mRNA and protein expression. This mechanism was observed in all the cell lines with variable responses to the combination of GSK3 and PARP inhibition. In contrast, depletion of GSK3α displayed no significant effect on PARPi sensitivity and HR repair. We also observed that combined GSK3β and PARP inhibition induced markers of replication stress, specifically p-Chk1 and p-RPA32 (S33, S4/8), and resulted in a high frequency of anaphase bridge formation. These data also showed that this combination led to mitotic spindle defects and induced G2/M cell cycle arrest. Therefore, we proposed that the mechanism of the synergistic interaction between GSK3i and PARPi may be in part due to replication stress and mitotic defects.

The previous study has shown that Wnt3a/GSK3β/Slug/Snail axis controlled EMT programs while coordinately regulating BRCA1 expression in breast cancer. Expressing low levels of BRCA1 co-express either nuclear Snail or Slug was found in the majority of triple-negative breast cancer (TNBC) patients^58^. However, the functional impact of Slug/Snail-dependent BRCA1 repression remains unclear. Our data confirmed that Slug and Snail played a pivotal role in Wnt3a/GSK3β-dependent BRCA1 expression in HCT-15 cells. Moreover, we also revealed a possibility of using GSK3i to sensitize PARPi, thereby expanding the benefits of PARPi to CRC. Although the protein expression of BRCA1 was almost completely abrogated, while the mRNA level of *BRCA1* only decreased to ~50% upon GSK3β inhibition and depletion, suggesting the involvement of other possible mechanisms. Therefore, the mechanism responsible for the suppression of BRCA1 expression by GSK3β remains to be further clarified.

Interestingly, this new combination strategy is likely more effective in BRCA2 deficient and BRCA proficient cancers than BRCA1-related cancers. In this study, we identified a strong synergistic inhibitory effect of GSK3β inhibition and PARP inhibition on all tested colon cancer. However, the combinations exerted a weaker synergistic effect against *BRCA1*-mutated cancers (e.g., HCC1937 and UWB1.289 cells). Compared with UWB1.289 cells, the BRCA1-proficient UWB1.289 + BRCA1 cells (UWB1.289 complemented with wild-type *BRCA1*) showed re-sensitive to the combination. The data also revealed that the synergistic effect of GSK3i and PARPi depended on BRCA1 in both HCT-15 and RKO cells. The selectivity of this new combination strategy needs to be further confirmed and clarified.

Collectively, our data provide a mechanistic understanding of combined PARP and GSK3 inhibition in CRC cells. Pharmacological and genetic studies suggested that loss of GSK3β activity impaired HRR efficacy, suppressed BRCA1 mRNA and protein levels, and substantially sensitized cells to PARPi and Top I inhibitors in replication-dependent DSBs lesions. Our study implies that GSK3β is an important modulator of HRR. Notably, GSK3i may be combined with PARPi-based treatments in a wider population of CRC patients.

## Supplementary information

Supplementary figures

Supplementary figure legend

Supplementary table
