# Supplementary figures and images for: Glycogen synthase kinase 3β inhibition synergizes with PARP inhibitors through the induction of homologous recombination deficiency in colorectal cancer
Source: Cell Death Dis. 2021 Feb 15;12(2):183. doi: 10.1038/s41419-021-03475-4 (PMC7884722; doi:10.1038/s41419-021-03475-4)

Figure S1


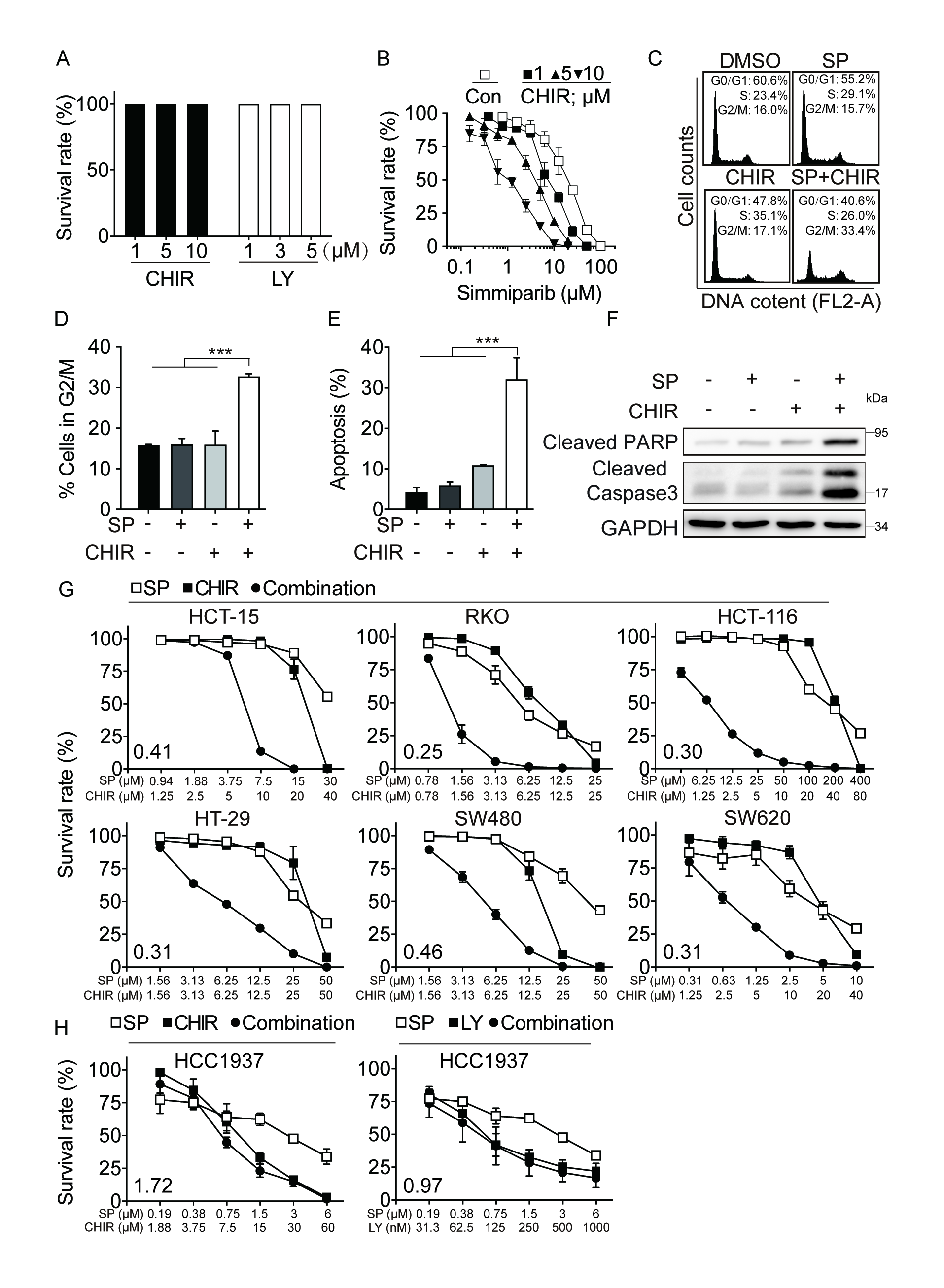


Figure S2


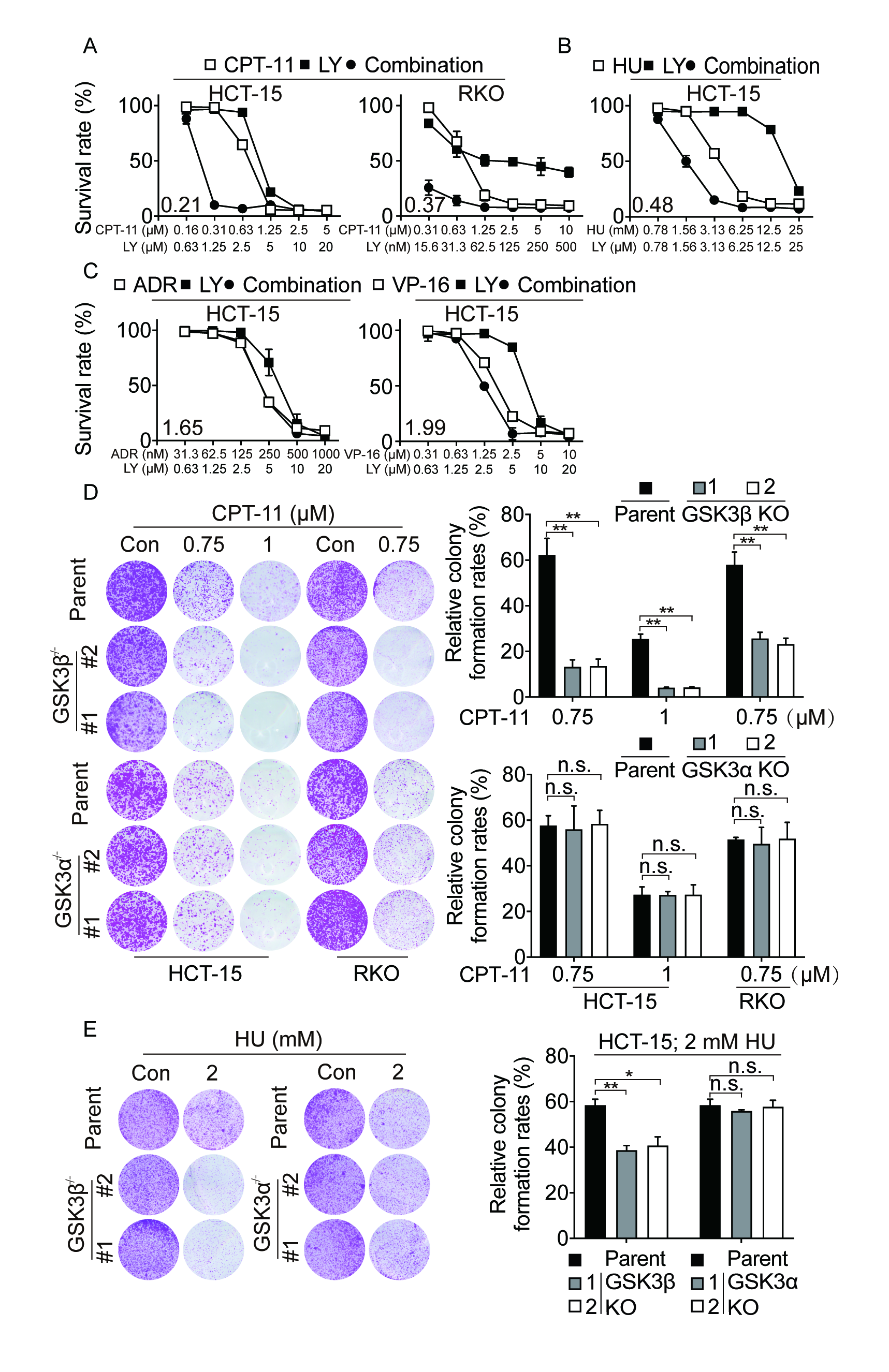


Figure S3


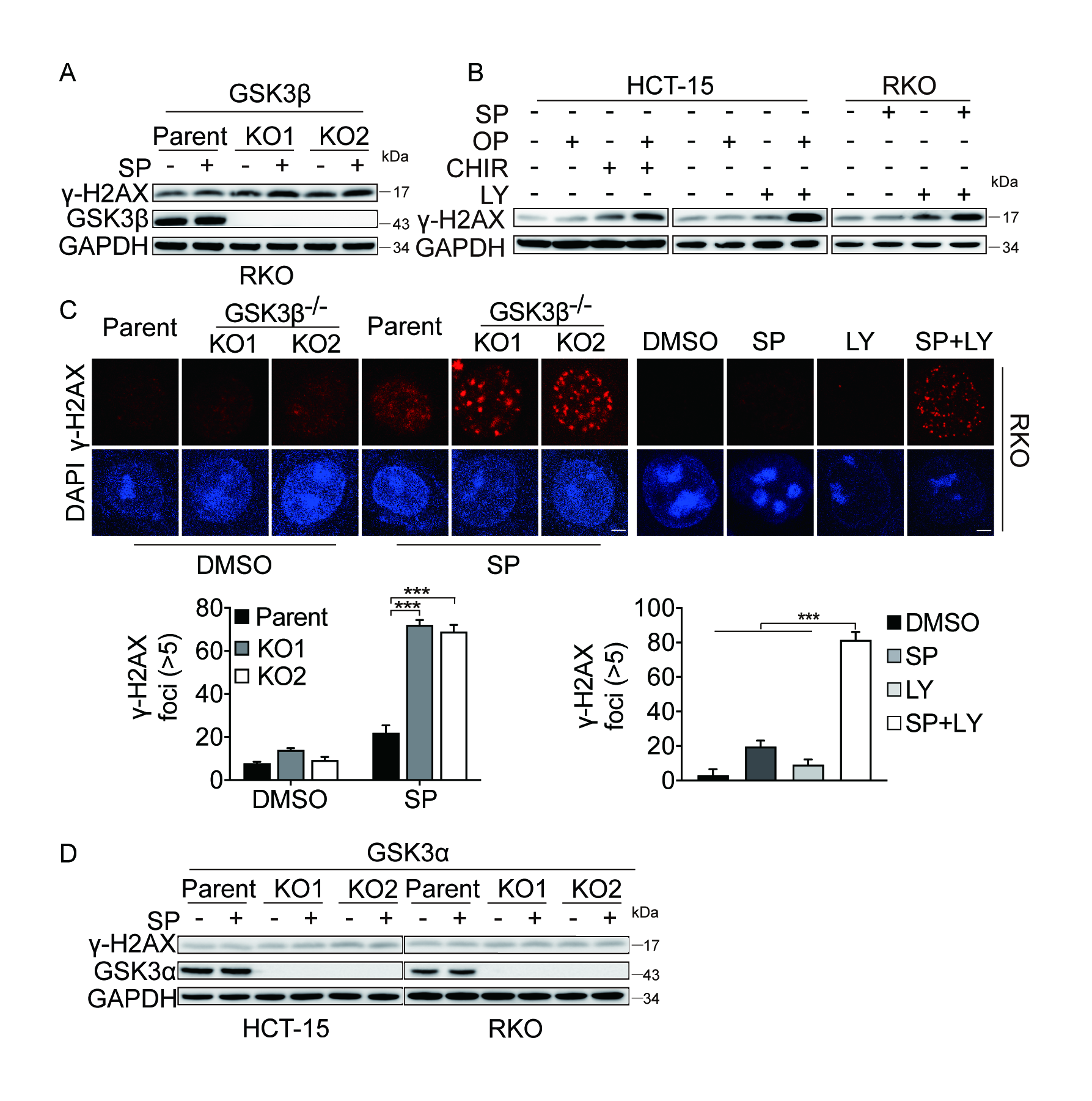


Figure S4


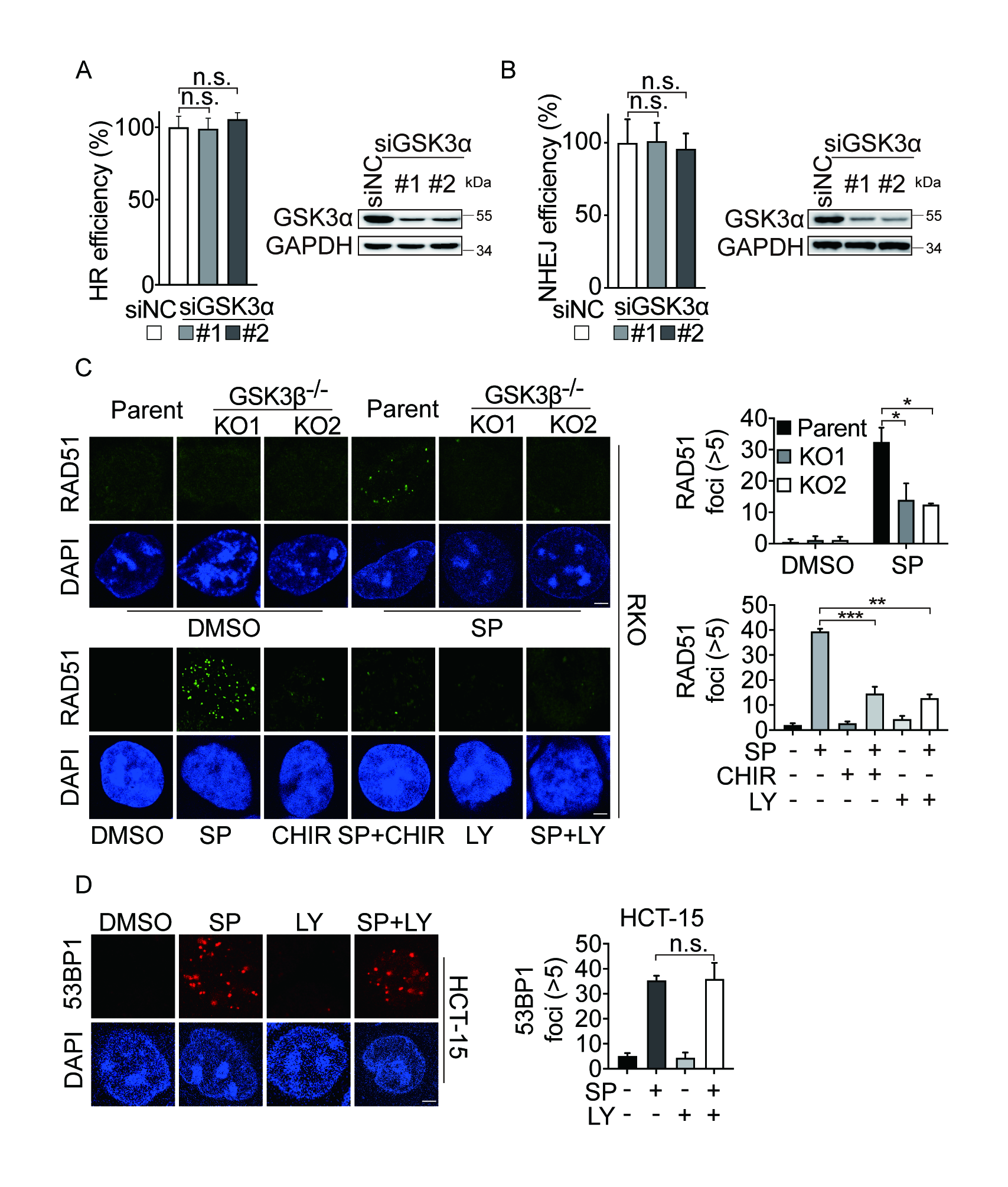


Figure S5


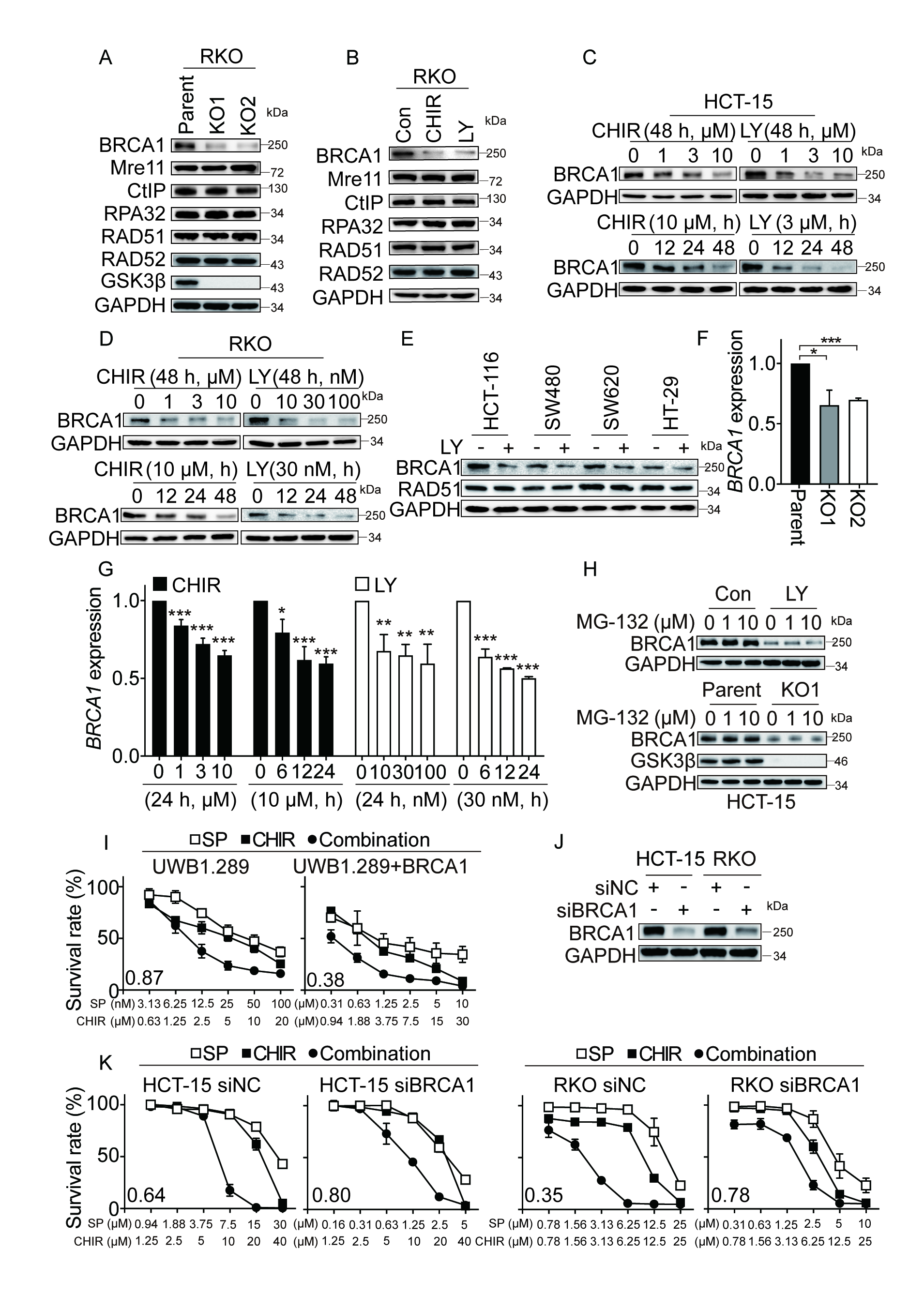


Figure S6


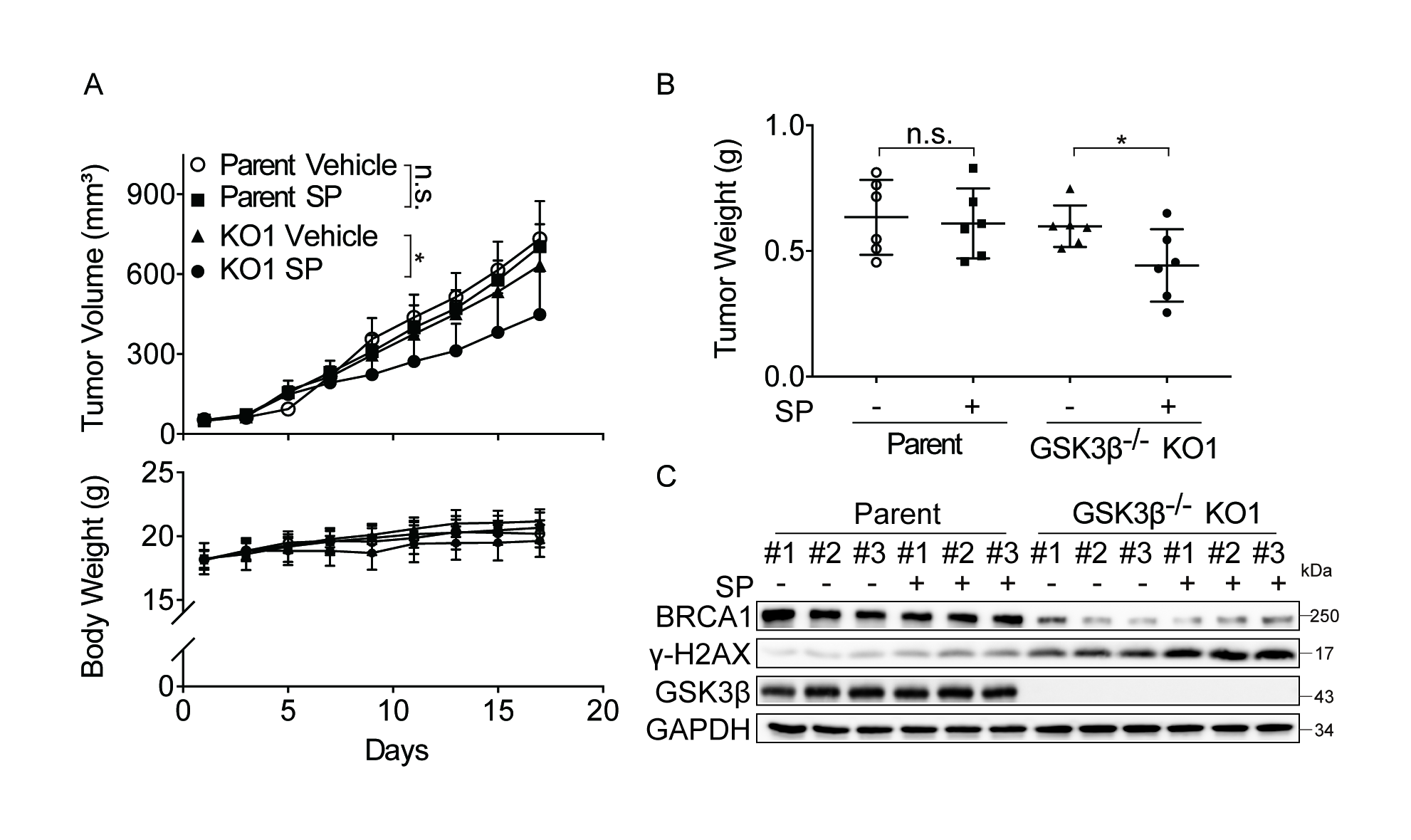

Supplement: Supplementary file 1 — Supplementary figures [file 41419_2021_3475_MOESM1_ESM.docx]
