# Supplementary material for: Glycogen synthase kinase 3β inhibition synergizes with PARP inhibitors through the induction of homologous recombination deficiency in colorectal cancer
Source: Cell Death Dis. 2021 Feb 15;12(2):183. doi: 10.1038/s41419-021-03475-4 (PMC7884722; doi:10.1038/s41419-021-03475-4)
Supplement: Supplementary file 2 — Supplementary figure legend [file 41419_2021_3475_MOESM2_ESM.docx]

**Supplementary figure legends**

**Figure S1. GSK3** **inhibition sensitizes CRC cells to PARPi**

**A,** Survival rate of HCT-15 cells treated with indicated CHIR99021 HCl (CHIR) or LY2090314 (LY). **B,** Dose-response curves of HCT-15 cells treated with indicated concentration of simmiparib (SP) with or without the CHIR99021 HCl (CHIR: 1, 5 and 10 μM) for 7 d. Data are from three independent experiments and expressed as mean ± SD**. C** and **D,** G2/M arrest induced by single agent or the indicated combination in HCT-15 cells was determined by FACS. Cells were treated with 5 μM simmiparib (SP), 10 μM CHIR99021 HCl (CHIR) or the indicated combination for 48 h and then subjected to FACS analysis. **(C),** Representative histograms are shown. **(D),** Percentage of cells in the G2/M phase expressed as mean ± SD from three independent experiments are shown. (****p <* 0.001, one-way ANOVA). **E,** Apoptosis induced by single agent or the indicated combination in HCT-15 cells. Cells were treated with 5 μM simmiparib (SP), 10 μM CHIR99021 HCl (CHIR) or a combination for 72 h and then analyzed using annexin V-FITC-PI-staining-based flow cytometry. Percentage of apoptotic cells in the expressed as mean ± SD from three independent experiments are shown. (****p <* 0.001, one-way ANOVA). **F**, Apoptosis-related proteins in HCT-15 cells treated with single agent or the indicated combination detected by western blotting. **G**, GSK3 inhibitor, CHIR99021 HCl (CHIR), sensitized a panel of cells to the PARPi, simmiparib (SP). The survival fraction and the average of CI values are shown from three independent experiments. **H**, Dose-response curves of simmiparib (SP) or GSK3i (CHIR, CHIR99021 HCl; LY, LY2090314) alone or combined in HCC1937 cell line treated with varying concentrations of the SP and GSK3i for 7 d. The survival fraction and the average of CI values are shown from three independent experiments.

**Figure S2. GSK3β depletion selectively sensitizes cancer cells to PARPi, Top I inhibitor and hydroxyurea**

**A** -**C**, Dose-response curves of the indicated agents alone or a combination in HCT-15 cells treated with varying concentrations for 7 d. CPT-11, irinotecan; ADR, Adriamycin; VP-16, etoposide; LY2090314, LY; Hydroxyurea, HU. The survival fraction and the average of CI values are shown from three independent experiments. **D** and **E** HCT-15 or RKO (parent) cells and their GSK3β-depleted or GSK3α-depleted single clone (KO1 and KO2) cells were treated with irinotecan (CPT-11; **D**) or hydroxyurea (HU; **E**) for 7 d. Cell viability was measured using colony formation assay. The IC_50_ values are expressed as mean ± SD from three independent experiments. (**p <* 0.05, ***p* < 0.01, n.s. not significant, *t*-test).

**Figure S3. Combined GSK3β and PARP inhibition increases DNA damage in CRC cells**

**A**, Western blot analysis of γ-H2AX in RKO cells (parent) or their GSK3β-depleted single clone (KO1 and KO2) cells treated with 5 μM simmiparib (SP) for 48 h. **B**, Western blot analysis of γ-H2AX in HCT-15 cells treated with 5 μM olaparib, GSK3i (10 μM CHIR99021 HCl or 5 μM LY2090314), or a combination for 48 h or in RKO cells treated with 5 μM simmiparib, 30 nM LY2090314, or a combination for 48 h. OP, Olaparib; SP, simmiparib; CHIR, CHIR99021 HCl; LY, LY2090314. **C,** Representative images of γ-H2AX foci in RKO (parent) and their GSK3β-depleted single clone (KO1 and KO2) cells treated with 5 μM simmiparib (SP, left panel) or HCT-15 cells following treatment with 5 μM simmiparib (SP), 30 nM LY2090314 (LY), or a combination for 48 h (Right panel). Nuclei were stained with DAPI. Scale bar: 2 μm. Cells that contained five or more γ-H2AX foci/nucleus were considered as γ-H2AX-positive cells. At least 50 cells were analyzed for each experiment and condition. All data are expressed as mean ± SD from three independent experiments. (Left: ****p* < 0.001, *t*-test; Right: ****p* < 0.001, one-way ANOVA). **D,** Western blot analysis of γ-H2AX in HCT-15 and RKO cells (parent) or their GSK3α-depleted single clone (KO1 and KO2) cells treated with 5 μM simmiparib (SP) for 48 h.

**Figure S4. GSK3β, but not GSK3α, is required for the homologous recombination repair of DSBs**

**A and B,** Quantification of the number of GFP-positive DR-U2OS cells (**A**) and NHEJ-Hela cells (**B**) treated with the indicated *GSK3α* siRNA. Data are expressed as mean ± SD from three independent experiments. (n.s. not significant, *t*-test). **C,** Representative images of RAD51 foci in RKO (parent) and their GSK3β-depleted single clone (KO1 and KO2) cells after treatment with 5 μM simmiparib (Upper) or RKO cells following treatment with 5 μM simmiparib, GSK3i (10 μM CHIR99021 HCl or 30 nM LY2090314), or a combination for 48 h (Lower). Nuclei were stained with DAPI. Scale bar: 2 μm. Cells that contained five or more RAD51 foci/nucleus were considered as RAD51-positive cells. All data are expressed as mean ± SD from three independent experiments. (**p* < 0.05, ***p* < 0.01, ****p* < 0.001, *t*-test). SP, simmiparib; CHIR, CHIR99021 HCl; LY, LY2090314. **D,** Representative images of 53BP1 foci in HCT-15 cells. Cells were treated with 5 μM simmiparib (SP), 5 μM LY2090314 (LY), or a combination for 48 h, and fixed for immunofluorescent staining of 53BP1 counterstained with DAPI. Scale bar: 2 μm. Cells that contained five or more 53BP1 foci/nucleus were considered as 53BP1-positive cells. At least 50 cells were analyzed for each experiment and condition. All data are expressed as mean ± SD from three independent experiments. (n.s., no significant, *t*-test).

**Figure S5 GSK3β depletion represses the expression of BRCA1**

**A,** Levels of DNA repair related proteins determined by western blotting in the parental RKO and their GSK3β-depleted single clone (KO1 and KO2) cells. **B,** Levels of DNA repair-related proteins determined by western blotting in RKO cells treated with 10 μM CHIR99021 HCl (CHIR) or 30 nM LY2090314 (LY). **C** and **D,** Change in BRCA1 protein levels in HCT-15 (**C**) and RKO (**D**) cells treated with GSK3i, CHIR99021 HCl (CHIR) and LY2090314 (LY), for indicated times and concentrations. **E,** Change in BRCA1 and RAD51 protein levels in indicated cells treated with 5μM LY2090314 (LY) for 48 h. **F** and **G**, mRNA level of *BRCA1* in the parental RKO and their GSK3β-depleted single clone (KO1 and KO2) cells after treatment with the indicated drugs was detected using qRT-PCR for indicated times and concentrations. CHIR, CHIR99021 HCl; and LY, LY2090314. (**F**: n = 3, **p* < 0.05, ****p* < 0.001, *t*-test; **G**: n = 3, **p* < 0.05, ***p* < 0.01, ****p*< 0.001, one-way ANOVA). **H**, Treatment with MG-132 failed to reverse the degradation of BRCA1 in the parental HCT-15 and their GSK3β-depleted single clone (KO1 and KO2) cells. The indicated cells were treated with either LY2090314 (LY) or MG-132 alone or in a combination for 24 h, and then the BRCA1 protein level was analyzed by western blotting. **I**, Effect of single agent and combination treatment on indicated cells viability for combinations of PARP inhibitor, simmiparib (SP), plus GSK3 inhibitor (CHIR99021, CHIR). Cell viability was measured by SRB assay. CI values were calculated using CompuSyn software with the Chou-Talalay equation, and average CI values are presented (CI < 1, synergism; CI = 1, additive effect; CI > 1 antagonism). Data are from three independent experiments and expressed as mean ± SD. **J**, Protein levels of BRCA1 detected by western blotting in siRNA-transfected HCT-15 and RKO cells. **K**, HCT-15 and RKO cells were transfected with siBRCA or siNC for 24 h and then followed by treatment of simmiparib (SP), CHIR99021 HCl (CHIR) or a combination for 7 d. The survival fraction and the average of CI values are shown from three independent experiments.

**Figure S6. PARP and GSK3β inhibition are synergistic *in vivo***

**A and B,** Mice (n = 6) bearing the indicated HCT-15 and their GSK3β-depleted KO1 xenograft tumors were dosed with or without simmiparib (SP, 50 mg/kg) every other day for 18 d. Tumor volume and body weight (**A**), were measured every other day, and tumor weight (**B**), was measured at the endpoint. (n = 6, **p* < 0.05, n.s., no significant, *t*-test). **C**, Protein levels of BRCA1, γ-H2AX, and GSK3β in the xenografts were determined using western blotting.
