# Supplementary material for: Glycogen synthase kinase 3β inhibition synergizes with PARP inhibitors through the induction of homologous recombination deficiency in colorectal cancer
Source: Cell Death Dis. 2021 Feb 15;12(2):183. doi: 10.1038/s41419-021-03475-4 (PMC7884722; doi:10.1038/s41419-021-03475-4)
Supplement: Supplementary file 3 — Supplementary table [file 41419_2021_3475_MOESM3_ESM.docx]

**Table S1. Compounds used in the combination screening**

| **Target** | **Catalog Number** | **Target** | **Catalog Number** | **Target** | **Catalog Number** |
| --- | --- | --- | --- | --- | --- |
| ALK | S8054 | ERK | S7709 | Aurora Kinase | S1133 |
|  | S7083 |  | S7554 |  | S2718 |
| BTK | S2680 | JNK | S4901 | BRD4 | S7305 |
|  | S7173 |  | S1460 |  | S7304 |
| c-Kit | S1018 | MEK | S2673 | DNA  Methyltransferase | S7276 |
|  | S1244 |  | S1036 |  | S1200 |
| c-Met | S1094 | p38 MAPK | S2726 | HDAC | S1030 |
|  | S2753 |  | S7215 |  | S1045 |
| EGFR | S7786 | Raf | S2807 | Histone  Acetyltransferase | S7641 |
|  | S1011 |  | S2220 |  | S7476 |
| FAK | S2013 | TOPK | S7648 | Histone  Methyltransferase | S3147 |
|  | S2890 |  | S7652 |  | S7062 |
| FGFR | S2801 | MNK | S6658 | Histone  Demethylase | S7070 |
|  | S1264 |  | S8257 |  | S7795 |
| FLT3 | S1526 | TAK1 | S8688 | Sirtuin | S1129 |
|  | S2158 |  | S8663 |  | S1541 |
| HER2 | S8362 | KLF | S8196 | Protease | S1013 |
|  | S7358 | JAK | S2851 |  | S2180 |
| IGF-1R | S8003 |  | S1378 | Akt | S7521 |
|  | S1124 |  | S2219 |  | S1078 |
| PDGFR | S2730 | Pim | S7041 | AMPK | S7953 |
|  | S2475 |  | S7104 |  | S7306 |
| PKC | S2791 |  | S2198 | ROCK | S1573 |
|  | S7208 | STAT | S4182 | DNA-PK | S2638 |
| Src | S1021 |  | S7977 |  | S1205 |
|  | S1006 | CDK | S1116 | GSK-3 | S7063 |
| Syk | S1533 |  | S7747 |  | S1263 |
|  | S8032 | Chk | S2626 | mTOR | S1039 |
| TGF-beta/Smad | S1067 |  | S1532 |  | S7035 |
|  | S7223 | PLK | S1485 | PDK | S1274 |
| Casein Kinase | S7642 |  | S1109 |  | S1275 |
| VEGFR | S1119 | ATR | S7050 | PI3K | S1065 |
|  | S1046 |  | S8007 |  | S1072 |
